# Diversity of Conopeptides and Conoenzymes from the Venom Duct of the Marine Cone Snail *Conus bayani* as Determined from Transcriptomic and Proteomic Analyses

**DOI:** 10.3390/md19040202

**Published:** 2021-04-03

**Authors:** Rajesh Rajaian Pushpabai, Carlton Ranjith Wilson Alphonse, Rajasekar Mani, Deepak Arun Apte, Jayaseelan Benjamin Franklin

**Affiliations:** 1 Centre for Molecular and Nanomedical Sciences, Centre for Nanoscience and Nanotechnology, Sathyabama Institute of Science and Technology, Chennai 600119, Tamil Nadu, India; jeshran@sathyabama.ac.in (R.R.P.); carltonranjith@sathyabama.ac.in (C.R.W.A.); drmrajasekar.irc@sathyabama.ac.in (R.M.); 2 Department of Marine Conservation, Bombay Natural History Society, Hornbill House, Dr. Sálim Ali Chowk, SBS Road, Mumbai 400 001, Maharashtra, India; da.apte@bnhs.org

**Keywords:** *Conus*, conotoxin, conopeptide, conoenzyme, transcriptome, mass spectrometry

## Abstract

Marine cone snails are predatory gastropods characterized by a well-developed venom apparatus and highly evolved hunting strategies that utilize toxins to paralyze prey and defend against predators. The venom of each species of cone snail has a large number of pharmacologically active peptides known as conopeptides or conotoxins that are usually unique in each species. Nevertheless, venoms of only very few species have been characterized so far by transcriptomic approaches. In this study, we used transcriptome sequencing technologies and mass spectrometric methods to describe the diversity of venom components expressed by a worm-hunting species, *Conus bayani*. A total of 82 conotoxin sequences were retrieved from transcriptomic data that contain 54 validated conotoxin sequences clustered into 21 gene superfamilies including divergent gene family, 17 sequences clustered to 6 different conotoxin classes, and 11 conotoxins classified as unassigned gene family. Seven new conotoxin sequences showed unusual cysteine patterns. We were also able to identify 19 peptide sequences using mass spectrometry that completely overlapped with the conotoxin sequences obtained from transcriptome analysis. Importantly, herein we document the presence of 16 proteins that include five post-translational modifying enzymes obtained from transcriptomic data. Our results revealed diverse and novel conopeptides of an unexplored species that could be used extensively in biomedical research due to their therapeutic potentials.

## 1. Introduction

Marine invertebrates are tremendous resources for bioactive molecules [1]. In particular, predatory cone snails possess a vast array of such molecules that include a variety of peptides and proteins that target various cell channels and receptors [2,3,4]. These venom components are expressed in the venom ducts of cone snails and usually injected into potential prey or predators via a hollow radular tooth [5]. Conotoxins are venom peptides comprised of between six and 50 amino acid residues and consist of linear molecules or peptides with multiple disulfide bonds among cysteines [6,7]. The pharmacological properties of cone snail venom peptides have triggered the extensive exploration of their sequences, structures, and biological targets [8]. The diversity of conotoxins is so enormous that, hitherto, about 2920 mature peptides and 2804 precursor sequences, belonging to 29 different gene superfamilies (A, B1, B2, B3, C, D, E, F, G, H, I1, I2, I3, J, K, L, M, N, O1, O2, O3, P, Q, S, T, V, Y, and Conodipine) [9] of conotoxins, have been identified [6,10,11,12,13]. Apart from these superfamilies, 15 temporary gene superfamilies identified in early divergent clade species have also been classified [10]. According to ConoServer on Sunday, 28 March 2021, there are 31 types of cysteine frameworks (I to V, VI/VII, VIII to XXX, XXXII and XXXIII) and 12 pharmacological families (α-, γ-, δ-, ε-, ι-, κ-, μ-, ρ-, σ-, τ-, χ- and ω-families) are so far described [10]. The widespread diversity of the structure, pharmacological targets, cysteine framework, and disulfide connectivity of conotoxins acts as a source of potential novel bioactive peptides [4,6,14].

Conotoxins are encoded by genes that contain up to several introns and are processed from a prepropeptide precursor (usually 50–110 amino acids) [15]. The prepropeptide precursor includes an N-terminal hyper-conserved signal peptide region (usually 19–27 amino acids), a conserved pro-region (usually 20–40 amino acids), and a C-terminal hypervariable mature toxin region (usually 6–50 amino acids) [11]. Conotoxins may also have a diversity of post-translational modifications. These molecules target a variety of ion channels; G-protein coupled receptors and neurotransmitter transporters with a high degree of specificity and affinity, characteristics that make them useful as tools in neuroscience research and potential therapeutic agents. Initially, mass spectrometry analysis and Edman degradation methods were used to study the amino acid sequences of venom peptides [16]. Conotoxins undergo multiple types of modification after translation catalyzed by post-translation modifying enzymes [17]. Currently, transcriptomics has emerged as a valuable tool to characterize the diversity of peptides expressed by individual snails and has increased the pace of finding novel conotoxins in recent years [18,19,20,21]. Although proteomic analyses provide details concerning post-translational modifications (PTM), they fail to address the difference between amino acids with isobaric masses. Nonetheless, transcriptome data lack information on post-translational modifications but demonstrate the translated amino acid sequence. Transcriptomics coupled with mass spectrometry plays a vital role in identifying profiles of venom diversity including the PTMs present in mature peptides [22,23].

Recent applications of high-throughput sequencing technologies to characterize the transcriptomes of the venom duct of cone snails have revealed new and novel cysteine patterns in several conopeptide gene superfamilies [18,22]. High-throughput sequencing can achieve higher sequencing depth and greater coverage of transcriptome so that even rare transcripts with low expression levels can be identified [15,24], providing a precise resolution of the conopeptide diversity in cone snails [21,25]. Hence, a comprehensive study of the peptide repertoire of lesser-known clades is vital for identifying toxins that are clade-specific and could result in the identification of new conopeptides, novel cysteine frameworks, and new gene superfamilies. The discovery of new conopeptides and identification of novel structures may lead to potential sources of drug candidates with therapeutic applications and for a better understanding of the diversity of conopeptides [20,26]. 

**Figure 1:**
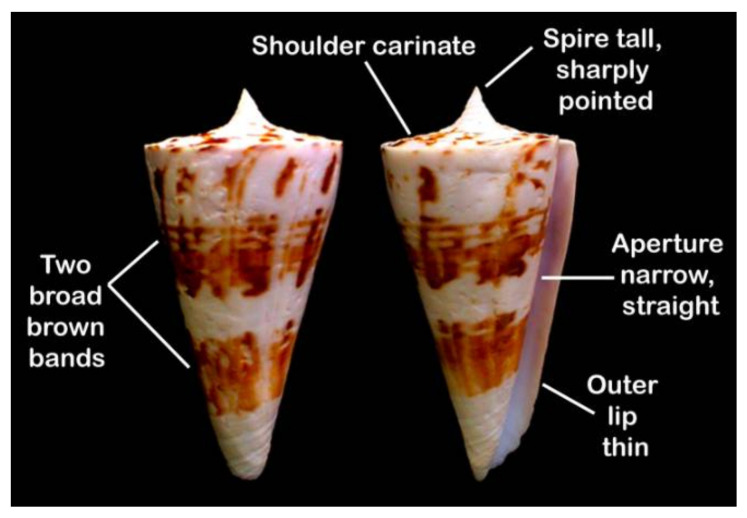
*Conus bayani* Jousseaume, 1872, key identification characters are annotated.

*Conus bayani* (Figure 1) is a vermivorous cone snail found in the north-west Indian Ocean from the central Red Sea to Somalia with discontinuous distribution. One set of population occurs from the south-east coast of India to Sri Lanka [27]. It occurs at depths from 20 to 100 m on the sandy substratum. Because these snails inhabit deep waters, aspects of their biology and ecology are poorly known. Currently, their venom profile too is largely unknown. Here, we characterized the diverse venom components of *C. bayani* using transcriptome analysis by Illumina sequencing technologies and proteomic analysis by mass spectrometry. In addition to novel conopeptide sequences, cysteine patterns, and PTMs, we present unique enzyme data obtained from transcriptomic data that include post-translational modifying enzymes.

## 2. Results

### 2.1. Transcriptome Sequences

Conotoxin transcriptome raw reads of *C. bayani* were deposited in the NCBI-SRA (Sequence Read Archive) database with project ID PRJNA704767. Post experimental analysis involves the pre-processing of the raw reads, adapter trimming, B-trimming, and low-quality end trimming. The de novo assemblies were constructed with Trinity, v2.2.0. The total number of contigs generated in SO_5664 was 112,133 with a maximum transcript length of 16,926 bp and in SO_5664_COGs, there were 99,351 with a maximum transcript length of 20,234 bp. The transcripts were generated and annotated with the molluscan database, more specifically with *Lottia gigantea* (owl limpet) and *Crassostrea gigas* (Pacific oyster). Further, more specific annotations were made with all available conotoxins within the databases.

**Figure 2:**
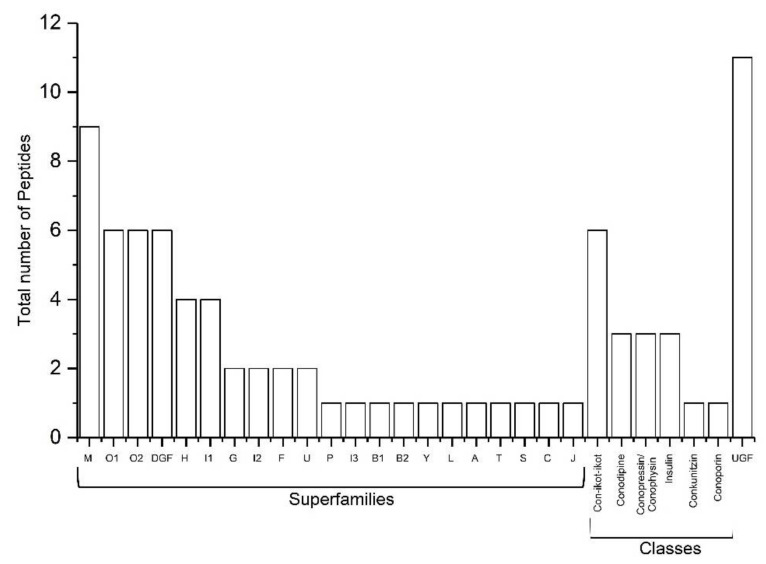
Total number of peptides identified from *C. bayani* venom duct transcriptome analysis is classified under different conotoxin superfamilies. UGF—unassigned gene family; DGF—divergent gene family.

**Figure 3:**
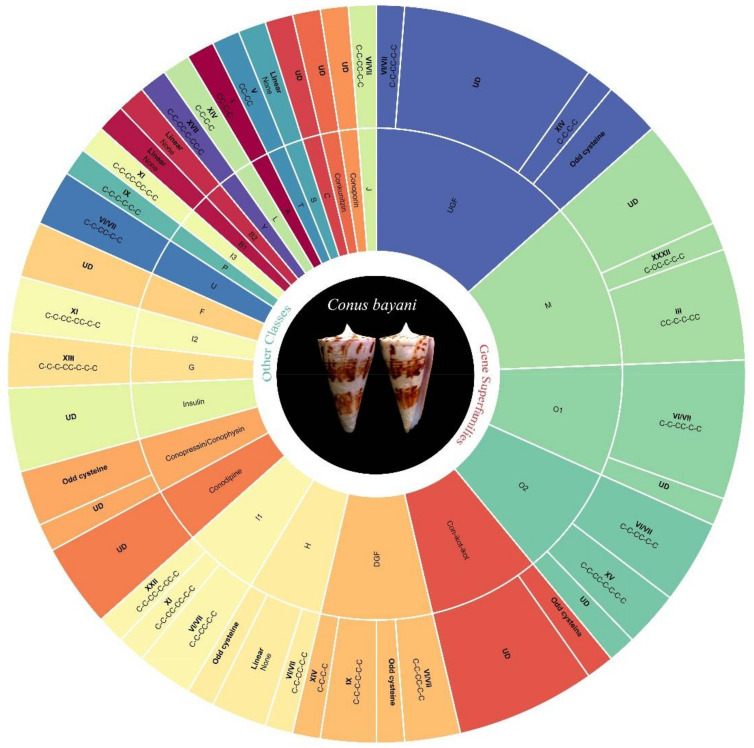
Graphical representation of the total number of peptides identified from *C. bayani* venom duct transcriptome analysis as classified under different conotoxin classes and cysteine patterns. UGF—unassigned gene family; DGF—divergent gene family.

In total, 82 conotoxin sequences were retrieved from transcriptomic data that contain 54 validated conotoxin sequences clustered into 21 gene superfamilies (O1, O2, M, H, G, P, I1, I2, I3, F, B1, B2, Y, L, A, T, U, S, J, C, and divergent gene family (DGF) [28]) and 17 others in six other different conotoxin classes (Conkunitzin, Conodipine, Conopressin/Conophysin, Con-ikot-ikot, Conoporin, and Insulin (Supplementary Table S1). In addition, 11 of them were not assigned to any superfamily were given here as UGF (undefined gene family [28]) M-superfamily accounted for the highest proportion of the known superfamily followed by O1, O2, and DGF (Figure 2 and Figure 3). A total of 12 known cysteine frameworks were also described. The maximum proportion of cysteine framework observed was VI/VII (C-C-CC-C-C). In addition, seven odd cysteine patterns were also discovered. We constructed 24 peptide sequences using mass spectrometry, in that 19 completely overlapped (Table 1; Sl. No. 1–19) with the conotoxin sequences obtained from transcriptome analysis (Table 1). Apart from these, we also identified 16 enzymes and proteins (Table 2) that include five post-translational modifying enzymes (PTM enzymes) from this study (Table 3).

A total of six O1-superfamily conotoxins were identified and among them, five conotoxins exhibited the VI/VII cysteine framework. Six O2-superfamily conotoxins were determined in which two exhibited the XV framework, three were of the VI/VII type, and one exhibited a cysteine framework with four cysteines that have not been described previously. A total of nine M-superfamily conotoxins were identified that exhibit different cysteine frameworks (four with III, one with XXXII, and four with previously undescribed frameworks). Four H-superfamily conotoxins were identified; these include two linear conotoxins, one with framework VI/VII, and another (“conotoxinba_contig_26”) which included an odd number of cysteines. Two peptides with cysteine framework XIII were identified and categorized as G-superfamily conotoxins. Seven conotoxins were categorized as members of the I-superfamily. Four of these conotoxins (one with XI, two with VI/VII, and one with XXII with framework) represent I1-superfamily conotoxins. The three others belong to framework XI and are categorized as I2 and I3-superfamilies. Two linear conotoxins were identified which represent B1 and B2-superfamily conotoxins. Two more conotoxins with unknown cysteine frameworks were identified and categorized as F-superfamily conotoxins. Single variants of Y, L, A, and T-superfamily conotoxins were found in the transcriptome data that exhibited XVII, XIV, I, and V cysteine frameworks, respectively. Two conotoxins with VI/VII framework were categorized as U-superfamily conotoxins. One linear S-superfamily conotoxin and one single disulfide-bonded C-superfamily conotoxin (contulakin) were also found. Six conotoxins that appear to represent a DGF (divergent gene family) divergent group were also detected. Among these, two possessed the VI/VII cysteine framework, one exhibited the XIV framework, two exhibited the IX framework, and one exhibited an odd number of cysteines. We could not place 11 conotoxins into any previously described superfamilies denoted as unassigned gene family (UGF). These include two conotoxins (one with XIV and the other with VI/VII framework) and nine others with no previously described cysteine framework among them two are odd number containing cysteines. Several conotoxins (ba_contig_26, ba_contig_53, ba_contig_54, ba_contig_59, ba_contig_68, ba_contig_76, and ba_contig_82) differ from other conotoxins which contain odd number of cysteines. Figure 2 and Figure 3 shows the abundance of different conotoxins identified by transcriptome analysis and categorized under different gene superfamilies and classes. 

One of the peptide sequences with an odd number of cysteines (ba_contig_26) that we identified appears similar to a sequence characterized from *Conus lenavati* (Cln_H_1) which possess four cysteines [29]. However, the former sequence obtained from *C. bayani* had only three cysteines. Given the sequence from *C. lenavati*, the first cysteine in the *C. bayani* sequence was replaced by arginine.

Conopressin/conophysin conotoxins (ba_contig_53 and ba_contig_54) also possessed an odd number of cysteines with 17 and 19 cysteine residues, respectively. In addition, one con-ikot-ikot conotoxin (ba_contig_59) is comprised of 13 cysteine residues. A published sequence from *Conus tribblei* (Ctr_39_N) [30] also possesses 13 cysteines and is similar to our sequence. Another conotoxin categorised under DGF ba_contig_68 with 7 cysteines, ba_contig_76 and ba_contig_82 of UGF contained 3 and 7 cysteines, respectively, which could not be placed in any previously defined category. Nonetheless, the most similar sequence that we could find from published accounts (i.e., G003_VD_Con-ikot-ikot_precursor_conopeptide from *Conus geographus*) [31] is made of 14 cysteines.

On the other hand, while one of the sequences we identified (ba_contig_72) closely matched a sequence from *Conus miles* (Mi047) [22], the sequence from *C. bayani* possesses four cysteines while the one from *C. miles* has six. Furthermore, another sequence that resembles a contryphan (ba_contig_20) exhibits a single disulfide bond and multiple aspartic acid residues. A similar sequence from *C. tribblei* (Ctr_M_2G) [32] has three aspartic acid residues while the translated peptide of *C. bayani* has six consecutive aspartic acid residues proximal to CPWC.

### 2.2. Validation by Mass Spectrometry

De novo sequencing of peptides was manually performed using Compass Data Analysis software version 4.1. Chemically modified crude venom is used for the sequencing of various peptides from the venom of *C. bayani*. The fragmented daughter ions of each parent ion were carefully analyzed and sequenced following the de novo sequencing strategy.

**Figure 4:**
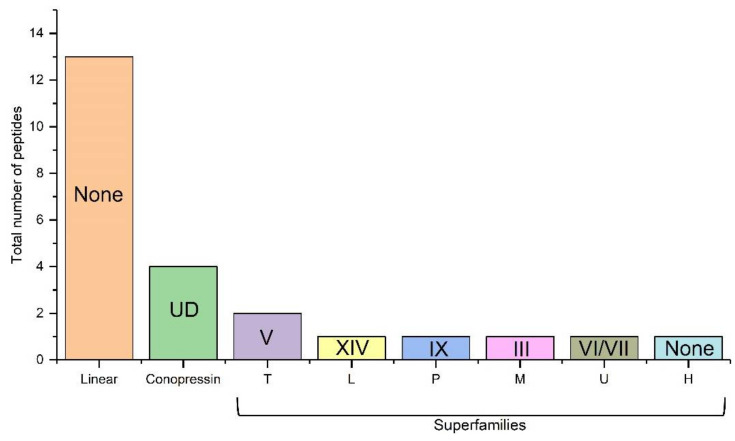
Total number of peptides identified as different conotoxin superfamilies using mass spectrometry analyses. UD—Undefined, None—No or absence of ‘Cys’ pattern, V, XIV, IX, III, VI/VII—Cysteine pattern.

A total of nine peptides with one disulfide to three disulfide bonds were manually sequenced (Table 1). Apart from these, a few linear conopeptides were also sequenced (Table 1). Mass spectrometric based sequencing resulted in four single disulfide conopressins (Table 1). Figure 4 illustrates the abundance of different gene superfamilies and classes of conotoxins identified by mass spectrometry based de novo sequencing.

The sequence of the conopressin peptides (Conopressin ba1a, Conopressin ba1b, Conopressin ba1c, and Conopressin ba1d) with mass numbers (m/z) 1023.5, 1081.3, and 1039.5, respectively, are presented in (Supplementary Figures S1–S4). These are similar to each other except for their PTMs. The peptide Conopressin ba1b had two glycine residues at the C-terminal position but looks like a premature form of a peptide prior to PTM processing to amidated Conopressin ba1a. The other peptide Conopressin ba1c is the same peptide as Conopressin ba1a except for the hydroxyproline residue in the seventh position of the peptide.

Two other peptides (ba5a and ba5b) are categorized as two disulfide-bonded T-Superfamily conotoxins (Table 1). The sequences of these two peptides ba5a and ba5b (Supplementary Figure S5) are nearly identical except that peptide ba5b possesses an extra serine residue in its first position. Another two di-sulfide containing conotoxin (ba14a) was classified as an L-superfamily conotoxin. The intact mass and their reduced and alkylated mass were detected (Supplementary Figure S6).

One sequence (ba9a) (3006.1) was classified as a P-superfamily conotoxin with three disulfide bonds. This conotoxin which possesses two different PTMs were unambiguously identified (Supplementary Figure S7). The fifth glutamic acid residue is modified to gamma carboxylic glutamic acid and hydroxyl proline in the 14th and 18th positions. This is the first P-superfamily conotoxin with a modified proline residue (hydroxyl proline) in all of the 14 P-superfamily conotoxin reported in Conoserver [10,12].

A peptide with three disulfide bonds were identified and sequenced from the venom of *C. bayani* is (ba3a) (1524.3) (Supplementary Figure S8). This was classified as an M1-superfamily conotoxin. Another three disulfide-bonded conotoxins sequenced by mass spectrometry is (ba-2281) (Supplementary Figure S9) was classified as U-Superfamily conotoxins which had undergone hyper hydroxylation of a proline residue to a hydroxyproline residue. This sequence also possesses five serine residues.

Apart from the peptides with disulfide bonds, we also observed several linear peptides from the venom of *C. bayani* (Table 1). Two of these sequences (ba1560.9 and ba1890.8) were categorized as members of the H-superfamily conotoxin (Table 1) (Supplementary Figure S10). From mass spectrometric sequencing of peptides from the N-terminal side, we found the delta mass difference of 111 D which corresponds to the presence of pyroglutamic acid residue. Sequences recovered from the transcriptome sequencing (ba_contig_23) confirmed the presence of glutamine residue in the N-terminal side in both the peptides. For the 19 peptides identified and/or sequenced in mass spectrometry, all were also identified from analyses of transcriptome data. Several other linear peptides were sequenced and named as ba 606.2, ba 818.3, ba 834.3, ba 731.3, ba 648.2, ba 745.7, ba 561.1, ba 416.9, ba 534.2, ba 774.7, ba 558.3, and ba 998.2 (Table 1, Supplementary Figures S11–S21), respectively. Except for ba 416.9, ba 534.2, ba 774.7, ba 558.3, and ba 998.2, all others were validated by transcriptome sequencing.

## 3. Discussion

Transcriptome sequencing has enabled the investigation and exploration of the huge diversity of conopeptides expressed by several *Conus* species [30]. Our study on the diversity of conopeptides and conoenzymes of *C. bayani* revealed 82 distinct conotoxin sequences that cluster into 21 gene superfamilies. Furthermore, 17 sequences cluster into six conotoxin classes. We obtained sequences of 16 different enzymes from the transcriptome data. While some of these enzymes may be involved in normal metabolic processes, five appear to represent PTM enzymes (Table 2). Post-translational modifications are one of the hallmarks of conotoxins [33]. All of the PTM events associated with these enzymes are evident in our results from peptide sequencing using mass spectrometry and transcriptome sequencing (Table 3).

Usually, M-superfamily conotoxins constitute the majority of conotoxins sequences in transcriptome analysis of venom duct of cone snails, followed by O-superfamily conotoxin [34,35,36]. Interestingly, *C. bayani* also exhibits this pattern in which M-superfamily conotoxin predominate in abundance, followed by O1 and O2-superfamilies of conotoxins, and subsequently by I1 and H-superfamily conotoxins. Nevertheless, other groups are relatively less abundant with just one or two unique sequences recovered from these classes. In mass spectrometric sequencing, linear conotoxins were most predominantly recovered.

Our efforts to couple transcriptomic and peptidomic strategies resolved the primary structure of mature conotoxins. The uncertainty of L/I/O and Q/K residues were clarified with the help of transcriptomic data. At the same time, several PTMs were solved using mass spectrometry-based peptidomic approaches. Therefore, our study describes the usefulness of both methods to obtain a robust data set. Another interesting finding is the occurrence of a contryphan-like peptide (contig: ba_contig_20) with a sequence that possesses six aspartate residues with a small cysteine loop at the C-terminal region that is similar to a peptide isolated from *Conus tribblei* which possessed only three aspartate residues before the CPWC loop [30]. Although very few studies have reported conotoxins with an odd number of cysteines [29,37], our study revealed the presence of seven such peptides.

## 4. Materials and Methods

### 4.1. Materials

Tris (2-carboxyethyl) phosphine (TCEP) was procured from Thermo Fisher Scientific, Inc. (Waltham, MA, USA). *N*-ethylmaleimide (NEM) was purchased from Sigma Aldrich (St. Louis, MO, USA). Analytical grade acetonitrile (ACN), methanol, and tri fluoro acetic acid (TFA) were obtained from Merck Ltd. (Bengaluru, Karnataka, India).

### 4.2. mRNA Extraction

Ten specimens of *Conus bayani* were collected as bycatch from the fishing nets of the fishing trawlers at Palayar (11°26′ N, 79°59′ E), Tamil Nadu, India. JBF was the taxonomy expert for cone snails in India and a biotechnologist who collected specimens and confirmed species identity [27]. The venom duct of each live specimen was dissected and flash-frozen in liquid nitrogen in the field immediately [38]. Frozen venom ducts were used to extract RNA.

### 4.3. RNA Quality Control

The tissue sample was lysed using 450 μL of buffer and homogenized in Micro Smash MS100 (TOMY SEIKO, Tokyo, Japan) using steel beads in the presence of liquid nitrogen. The lysate was centrifuged to remove cell debris and the supernatant was mixed with ethanol prior to loading onto RNeasy (Qiagen, Venlo, Holland). The remaining steps of the protocol were followed as per the manufacturer’s guidelines including on-column DNase (Qiagen) treatment. RNA was eluted in Nuclease free water (Ambion, Cat#AM9938). The concentration and purity of the RNA extracted was evaluated using Nanodrop Spectrophotometer 2000 (Thermofisher Scientific, Waltham, MA, USA). The integrity of the extracted RNA was analyzed on Agilent 2100 Bioanalyzer (Agilent, Santa Clara, CA, USA).

### 4.4. Transcriptome Library Preparation and Sequencing

The transcriptome sequencing library was prepared using NEBNext Ultra Directional RNA library prep kit (New England BioLabs, Ipswich, MA, USA) at Genotypic Technology Pvt. Ltd., Bangalore, India. Then, 200 ng of total RNA was taken for mRNA isolation by using NEBNext Poly(A) mRNA magnetic isolation module as per the manufacturer’s protocol. The isolated mRNA was subjected to fragmentation using magnesium ions at 94 °C for 8 min. The fragmented mRNA was subjected to random priming-based reverse transcription in the presence of Actinomycin D (Gibco, Life Technologies, Dun Laoghaire, Dublin, Ireland) followed by second-strand cDNA synthesis. The double-stranded cDNA was purified using HighPrep magnetic beads (Magbio Genomics Inc, Gaithersburg, MD, USA). Purified cDNA was end-repaired, adenylated and ligated to Illumina adapters as per NEBNext protocol. Adapter ligated cDNA was purified using HighPrep beads and subjected to 14 cycles of enrichment PCR in the presence of a specific barcode primer. Final libraries were purified using HighPrep magnetic beads and reconstituted in nuclease-free water. The libraries were quantified by Qubit dsDNA HS assay (Thermo Fisher Scientific, Waltham, MA, USA) and its fragment size distribution was analyzed on Agilent 2200 Tape Station (Agilent Technologies, Santa Clara, CA, USA). The libraries were sequenced with an Illumina NextSeq 500 sequencer (Illumina, San Diego, CA, USA) for 150 bp paired-end chemistry following the manufacturer’s procedure.

### 4.5. NGS Data Analysis

The raw data generated were quality checked using FastQC v0.11.5 [39]. Reads were pre-processed to remove adapter sequences and low-quality bases (<q30). Pre-processing of data was done with an in-house script. Processed reads were assembled using a graph-based approach in the Trinity v2.2.0 with default k-mer [40]. Trinity combines the overlapping reads of a given length and quality into longer contig sequences without gaps. The characteristic properties, including N50 length, average length, maximum length, and a minimum length of the assembled contigs were calculated. To assess the quality of the assembly, evaluation of read content approach was used. Assembled transcripts were annotated using a homology approach to assign functional annotation using BLAST against “Mollusca” data from the Uniprot database [41,42]. Transcripts were assigned with a homolog protein from other organisms if the match was found at e-value less than e-5 and minimum similarity greater than 30%. Pathway analysis was done by using KAAS Server [43]. Transcripts were annotated using the ConoServer database [10].

### 4.6. Crude Venom Extraction

Twenty frozen venom ducts were minced to short pieces and extracted with 20 mL of 50% acetonitrile. The extract was cold steeped for a week and the resulting mixture was filtered and the clear solution was lyophilized and stored in −20 °C until further use [38].

### 4.7. Mass Spectrometry Analysis

An aliquot of lyophilized crude venom (approximately 200 µg) was dissolved in a 50:50 solution of acetonitrile: water with 0.1% TFA. To 20 µL of the crude venom extract, TCEP (final concentration 20 mM) was added. The mixture was incubated at 37 °C for 1.5 h. To this reaction mixture, NEM was added to a final concentration of 40 mM and incubated at room temperature for 60 min. The reaction mixture was analyzed by LC-ESI-MS on a Bruker Daltonics (Bremen, Germany) Esquire 3000 Plus Ion-Trap Mass Spectrometer attached to an Agilent 1100 series HPLC system. The samples were infused into the mass spectrometer either by direct injection or through an HPLC column (Agilent Zorbax analytical C18 column, 150 × 4.6 mm, 5 µm, 90 Å pore size) and eluted using a binary gradient of water (0.1% TFA): acetonitrile (0.1% TFA) at a flow rate of 0.2 mL min-1 [44,45]. Data were acquired over a m/z range of 100–2000 in positive ion mode which enabled us to identify the number of disulfide-rich conopeptides and to establish the number of disulfides in each m/z species. Mass analysis were also carried out in MALDI Ultra-flex time of flight-mass spectrometer (BrukerDaltonics, Bremen, Germany) using a positive/negative-ionization mode in a 90 ns time delay, and a 25 kV accelerating voltage. The system utilizes a 50 Hz pulsed nitrogen laser, emitting at 337 nm. The ion source and the flight tube were kept at a pressure of about 7 × 10^−7^ mbar by turbo molecular pumps. The sample was prepared by dissolving 50% (*v*/*v*) of a saturated solution of matrix α-Cyano-4-hydroxycinnamic acid (CHCA), dihydroxybenzoic acid (DHB), and sinapic acid (SA) in ACN [44,45]. A standard peptide was used for external calibration. Full scan (from 500 to 10,000 *m*/*z*) was performed in linear mode.

### 4.8. Mass Spectrometry Data Analysis

Data were analyzed within the m/z range of 5000, as there were no signals observed or lack of ionization of peptides beyond the range for *C. bayani* venom. Data were analyzed using Flex-Analysis Software (1.3, Bruker Daltonics, Bremen, Germany). To 10 µL of the crude venom extract of reduced and alkylated natural venom, 2 µL of acetic anhydride was added, and the volume was made up to 20 µL with distilled water. The reaction mixture was incubated for 1 h at 25 °C and the product was analyzed by LC-ESI-MS as described above to identify the conopeptide sequences that had a free amino terminus and/or have lysine residues in the sequence. The acetylation reaction also enabled the distinction between lysine and glutamine residues. Auto MS(n) experiments (CID fragmentation) were performed to the reduced alkylated, reduced alkylated and acetylated, reduced alkylated, and esterified crude extract. The peptides eluting from the column were fragmented using nitrogen gas (CID fragmentation). The daughter ion spectra were analyzed to derive the sequence of the peptides. The derived peptide sequences were compared with known peptide sequences of conotoxins. Experimental LC-ESI-MS data was analyzed using Bruker Daltonics Data Analysis Version 4.1 (Bremen, Germany). MALDI-TOF data was analyzed using Bruker Daltonics flex Analysis Software (1.3, Bremen, Germany) [44,45]. The daughter ions generated from the multiply charged and singly charged ions of each peptides which are chemically modified (reduction and alkylation, acetylation and esterification) were carefully analyzed manually and the amino acid sequence of several peptides were sequenced.

## 5. Conclusions

In summary, this study explores the diversity of conotoxins and conoenzymes of the marine snail *Conus bayani*. A total of 82 conotoxin sequences were identified, most of which could be classified into 21 gene superfamilies and few sequences in six other gene superfamily classes. M-Superfamily accounted for the highest proportion of the total sequences obtained followed by O1 and O2 gene superfamilies. Eleven sequences were likely new and are placed in undefined group. In addition, 16 enzymes and proteins were observed in this study that includes five post-translational modifying enzymes (PTM enzymes). Further, 24 sequences were determined from mass spectrometry method. Of which, 19 sequences are exactly similar to the transcriptome data. This study also describes the usefulness of integrating transcriptome and proteome profiling to obtain a robust data. Although the biological functions and molecular targets of the conopeptides characterized from *C. bayani* still remain to be demonstrated in future studies, the diversity of the identified conopeptides in this species suggests the wide range of molecular targets and biological functions of these peptides. This is the first report into the diverse conopeptide repertoire of *C. bayani*.

## Figures and Tables

**Table 1:** List of peptides sequenced from *C. bayani* using mass spectrometry.

Sl. No	Name	Contig No.	Mass (M + H)	Tentative Sequence	Notes
1.	Conopressin ba 1a	ba_contig_53	1023.5	CYITNCPRG-NH2	Conopressin
2.	Conopressin ba 1b	ba_contig_53	1081.3	CYITNCPRGG	
3.	Conopressin ba 1c	ba_contig_53	1039.5	CYITNCORG-NH2	
4.	Conopressin ba 1d	ba_contig_54	881.2	CFLGNCLN	
5.	ba5a	ba_contig_44	1016.6	CCGSSNTGSCC-NH_2_	T-Superfamily
6.	ba5b	ba_contig_44	1103.3	SCCGSSNTGSCC-NH_2_	
7.	ba14a	ba_contig_42	1981.6	TLCPEHCTNGCNMDMTCI	L Superfamily
8.	ba9a	ba_contig_29	3006.1	VSCGgYCGDYGDCOSSCOTCTSNLLKCM	P-Superfamily
9.	ba3a	ba_contig_19	1524.3	TCCTACNIPPCKCCA	M-Superfamily
10.	ba-2281	ba_contig_45	2881.2	SSQSTCOYCQISCCOOAYCQOSGCRGP	U superfamily
11.	ba1560.9	ba_contig_23	1560.9	QNDHDVDESGHDIP	H-Superfamily LINEAR
12.	ba1890.8	ba_contig_23	1890.8	QNDHDVDESGHDIPFPS	
13.	ba 606.2	ba_contig_83	606.2	NSIWS	LINEAR
14.	ba 818.3	ba_contig_83	818.3	DPNSIWS	LINEAR
15.	ba 834.3	ba_contig_83	834.3	DONSIWS	LINEAR
16.	ba 731.3	ba_contig_83	731.3	DPNSIW	LINEAR
17.	ba 648.2	ba_contig_84	648.2	RSLWS	LINEAR
18.	ba 745.7	ba_contig_84	745.7	PRSLWS	LINEAR
19.	ba 561.1	ba_contig_84	561.1	RSLW	LINEAR
20.	ba 416.9	Not found	416.9	DEGP	LINEAR
21.	ba 534.2	Not found	534.2	FSGHS	LINEAR
22.	ba 774.7	Not found	774.7	KVL/IKATD	LINEAR
23.	ba 558.3	Not found	558.3	KVL/IKA	LINEAR
24.	ba 998.2	Not found	998.2	EGHDLPFPS	LINEAR

**Table 2:** List of enzymes and other proteins identified from *C. bayani* venom duct transcriptome analysis.

Sl. No	Contig No	List of Enzymes and Other Proteins
1.	ba_contig_85	78 kDa glucose-regulated protein
2.	ba_contig_86	Arginine kinase
3.	ba_contig_87	Conotoxin-specific protein disulfideisomerase (Cspdi)
4.	ba_contig_88	Cysteine-rich protein 1
5.	ba_contig_89	Cysteine-rich venom protein (CRVP) (Substrate-specific endoprotease Tex31)
6.	ba_contig_90	Cysteine-rich venom protein Mr30 (CRVP) (Cysteine-rich secretory protein Mr30) (GlaCrisp isoform 1/2/3) (Mr30-1/2)
7.	ba_contig_91	Cytochrome b
8.	ba_contig_92	Cytochrome c oxidase subunit 1
9.	ba_contig_93	Ferritin (EC 1.16.3.1) (Fragment)
10.	ba_contig_94	Glycoprotein hormone alpha-2 prepropeptide
11.	ba_contig_95	NADH-ubiquinone oxidoreductase chain 5 (EC 1.6.5.3)
12.	ba_contig_96	Peptidylglycine alpha-amidating monooxygenase (EC 1.14.17.3)
13.	ba_contig_97	Peptidyl-prolylcis-trans isomerase (PPIase) (EC 5.2.1.8)
14.	ba_contig_98	Prohormone-4 prepropeptide
15.	ba_contig_99	Protein disulfide isomerase
16.	ba_contig_100	Vitamin K-dependent gamma-glutamyl carboxylase

**Table 3:** Post-translational modifying enzymes identified from *C. bayani* venom duct transcriptome analysis.

Enzymes	Functions	Possible Modified Conotoxins
Conotoxin-specific protein disulfide isomerase (Cspdi)	Disulfide formation	CCGSSNTGSCC-NH_2_
Peptidylglycine alpha-amidating monooxygenase (EC 1.14.17.3)	Amidation	SCCGSSNTGSCC-NH_2_
Peptidyl-prolyl cis-trans isomerase (PPIase) (EC 5.2.1.8)	Cis-trans isomerization of proline imidic peptide bonds in oligopeptides	SSQSTCOYCQISCCOOAYCQOSGCRGP
Vitamin K-dependent gamma-glutamyl carboxylase	Gamma-carboxylation of Glutamic acid	VSCGgYCGDYGDCOSSCOTCTSNLLKCM
Prolyl 4-hydroxylase subunit alpha-2	Hydroxylation of Proline	SSQSTCOYCQISCCOOAYCQOSGCRGP

## Data Availability

https://www.ncbi.nlm.nih.gov/Traces/study/?acc=PRJNA704767 (accessed on 25 February 2021). Study: PRJNA704767. Sample: SO_5664_Snail (SAMN18055130). Experiment: CBA_Venom1 (SRX10167074). Run: SO_5664_Snail_R1.fastq.gz (SRR13781584).

## Supplementary Materials

The following are available online at https://www.mdpi.com/article/10.3390/md19040202/s1, Figure S1: Collusion induced fragmentation of Conopressin ba 1a from C. bayani illustrating arrangements of ‘y’ and ‘b’ ions from the parent ion (reduced and alkylated) 638.4 [M+H], Figure S2: Native reduced alkylated spectrum of conopressin ba 1a and Conopressin ba 1b showing 252 Da increase in mass confirming two cysteine residues, Figure S3: Spectrum showing all three native conopressin from the venom of C. bayani (Conopressin ba 1a, Conopressin ba 1b and Conopressin ba 1c), Figure S4: Spectrum showing native conopressin ba 1d from the venom of C. bayani, Figure S5: Collusion induced fragmentation of T superfamily conotoxin ba5b from C. bayani illustrating arrangements of ‘y’ and ‘b’ ions from the parent ion (reduced and alkylated) 804.3 [M+2H]+2, Figure S6: Native reduced alkylated spectrum of L superfamily conotoxin showing 504 Da increase in mass confirming four cysteine residues, Figure S7: Collusion induced fragmentation of P- superfamily conotoxin ba9a from C. bayani illustrating arrangements of ‘y’ and ‘b’ ions from the parent ion (reduced and alkylated) 1255.50 [M+3H]+3, Figure S8: Collusion induced fragmentation of M- superfamily conotoxin ba3a from C. bayani illustrating arrangements of ‘y’ and ‘b’ ions from the parent ion (reduced and alkylated) 1141.61 [M+2H]+2, Figure S9: Collusion induced fragmentation of U- superfamily conotoxin ba2281from C. bayani illustrating arrangements of ‘y’ and ‘b’ ions from the parent ion (reduced and alkylated) 1213.65 [M+3H]+3, Figure S10: Collusion induced fragmentation of H- superfamily conotoxin ba1560.9 from C. bayani illustrating arrangements of ‘y’ and ‘b’ ions from the parent ion 780.79 [M+2H]+2, Figure S11: Collusion induced fragmentation of linear conotoxin ba606.2 from C. bayani illustrating arrangements of ‘y’ and ‘b’ ions from the parent ion 606.19 [M+H], Figure S12: Collusion induced fragmentation of linear conotoxin ba818.3 from C. bayani illustrating arrangements of ‘y’ and ‘b’ ions from the parent ion 818.27 [M+H], Figure S13: Collusion induced fragmentation of linear conotoxin ba731.3 from C. bayani illustrating arrangements of ‘y’ and ‘b’ ions from the parent ion 731.28 [M+H], Figure S14: Collusion induced fragmentation of linear conotoxin ba648.2 from C. bayani illustrating arrangements of ‘y’ and ‘b’ ions from the parent ion 648.29 [M+H], Figure S15: Collusion induced fragmentation of linear conotoxin ba745.2 from C. bayani illustrating arrangements of ‘y’ and ‘b’ ions from the parent ion 745.27 [M+H], Figure S16: Collusion induced fragmentation of linear conotoxin ba561.1 from C. bayani illustrating arrangements of ‘y’ and ‘b’ ions from the parent ion 561.17 [M+H], Figure S17: Collusion induced fragmentation of linear conotoxin ba416.9 from C. bayani illustrating arrangements of ‘y’ and ‘b’ ions from the parent ion 416.99 [M+H], Figure S18: Collusion induced fragmentation of linear conotoxin ba534.2 from C. bayani illustrating arrangements of ‘y’ and ‘b’ ions from the parent ion 534.26 [M+H], Figure S19: Collusion induced fragmentation of acetylated linear conotoxin ba774.7 from C. bayani illustrating arrangements of ‘y’ and ‘b’ ions from the parent ion 859.24 [M+H], Figure S20: Collusion induced fragmentation of acetylated linear conotoxin ba558.3 from C. bayani illustrating arrangements of ‘y’ and ‘b’ ions from the parent ion 643.37 [M+H], Figure S21: Collusion induced fragmentation of linear conotoxin ba998.2 from C. bayani illustrating arrangements of ‘y’ and ‘b’ ions from the parent ion 998.29 [M+H], Table S1: Showing all conotoxins sequenced from next generation transcriptome analysis with their possible cysteine frameworks.
