# Supplementary material for: Diversity of Conopeptides and Conoenzymes from the Venom Duct of the Marine Cone Snail Conus bayani as Determined from Transcriptomic and Proteomic Analyses
Source: Mar Drugs. 2021 Apr 3;19(4):202. doi: 10.3390/md19040202 (PMC8066144; doi:10.3390/md19040202)
Supplement: Supplementary file 1 [file marinedrugs-19-00202-s001.pdf]

Diversity of conopeptides and conoenzymes from the venom duct of the marine cone snail *Conus bayani* as determined from transcriptomic and proteomic analyses

Rajesh Rajaian Pushpabai[1], Carlton Ranjith Wilson Alphonse [1], Rajasekar Mani [1], Deepak Arun Apte[2], Jayaseelan Benjamin Franklin [2*]

[1] Centre for Molecular and Nanomedical Sciences, Centre for Nanoscience and Nanotechnology, Sathyabama Institute of Science and Technology, Chennai, 600119, Tamil Nadu, India.
[2] Department of Marine Conservation, Bombay Natural History Society, Hornbill House, Dr. Sálim Ali Chowk, SBS Road, Mumbai 400 001, Maharashtra, India.
[2] Email: b.franklin@bnhs.org; benkutti@gmail.com
* Corresponding Author

Figure S1. Collision-induced fragmentation of Conopressin ba 1a from *C. bayani* illustrating arrangements of ‘y’ and ‘b’ ions from the parent ion (reduced and alkylated) 638.4 [M+H]

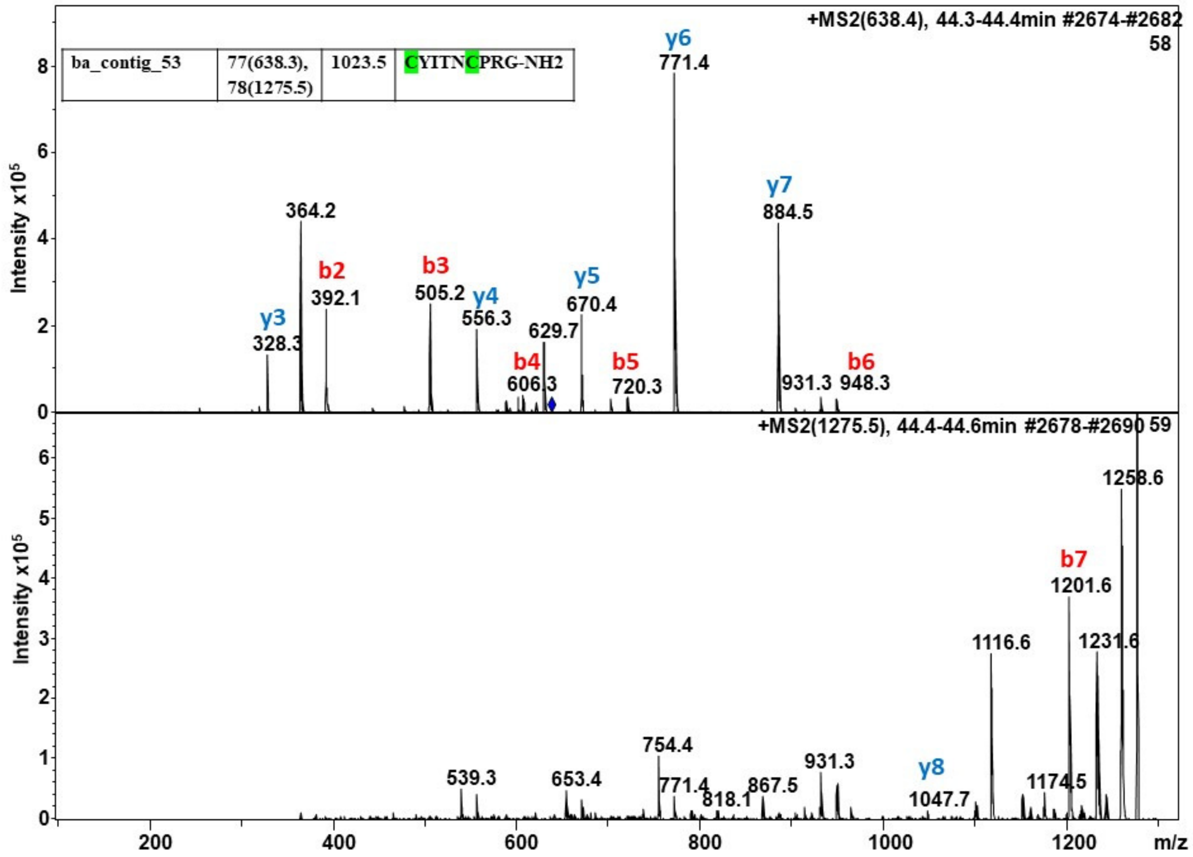

Figure S2. Native reduced alkylated spectrum of conopressin ba 1a and Conopressin ba 1b showing 252 Da increase in mass confirming two cysteine residues.

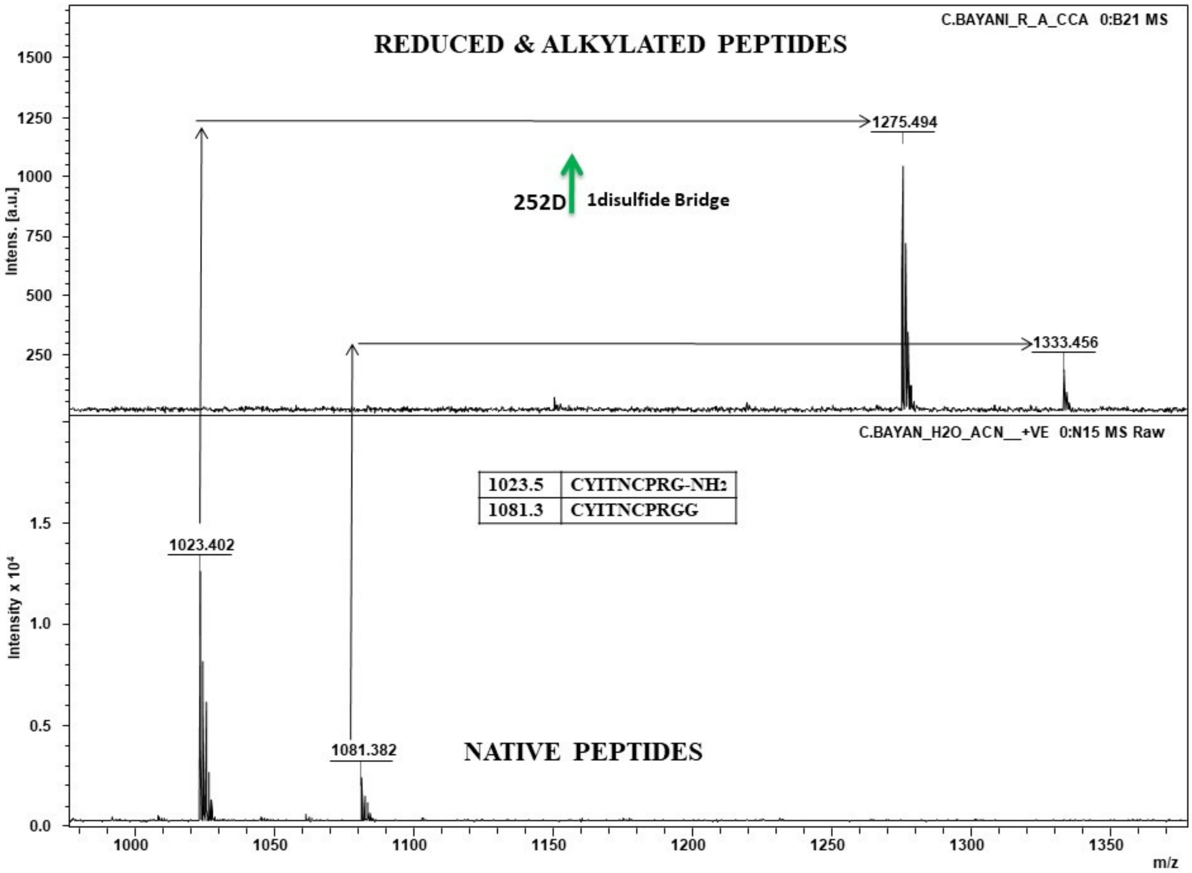

Figure S3. Spectrum showing all three native conopressin from the venom of *C. bayani* (Conopressinba 1a, Conopressinba 1b and Conopressinba 1c)

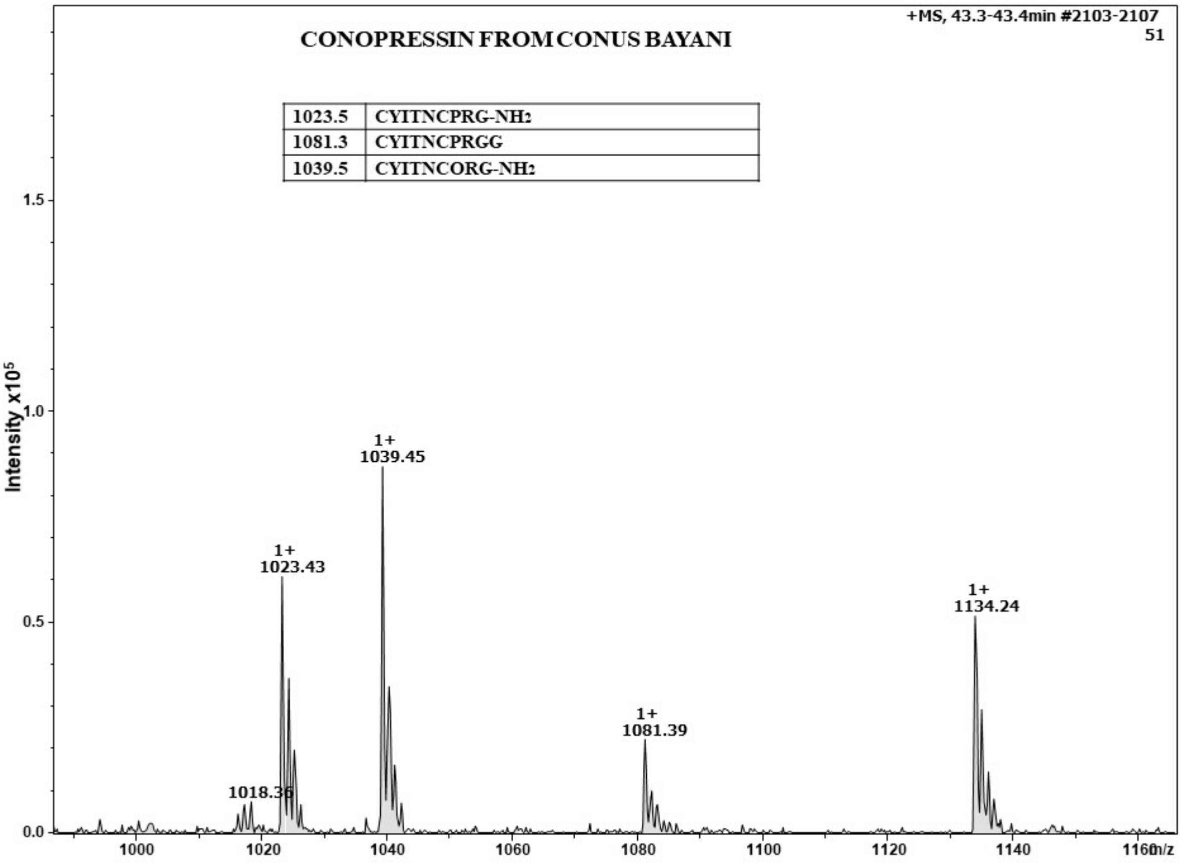

Figure S4. Spectrum showing native conopressin ba 1d from the venom of *C. bayani*

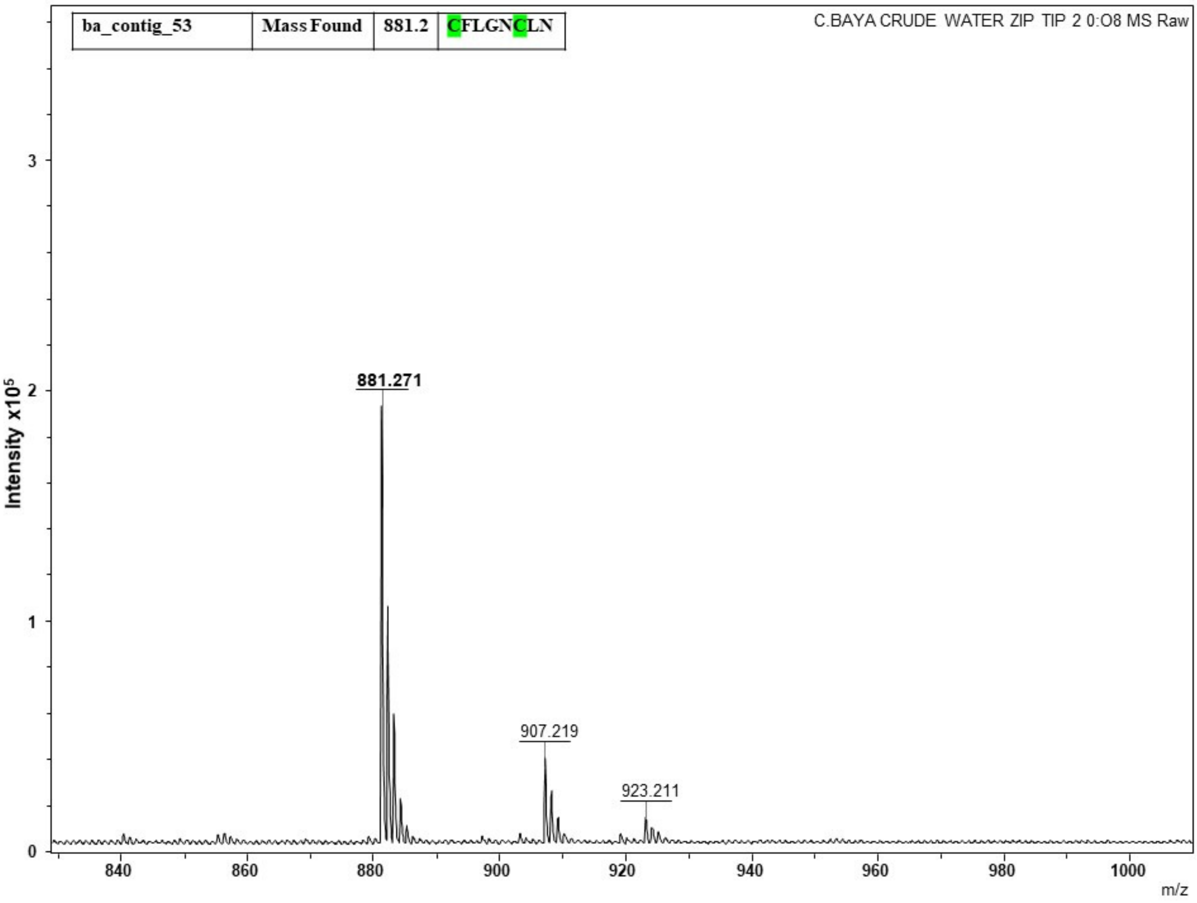

Figure S5 Collision-induced fragmentation of T superfamily conotoxinba5b from *C. bayani* illustrating arrangements of ‘y’ and ‘b’ ions from the parent ion (reduced and alkylated) 804.3 $[M+2H]^{+2}$

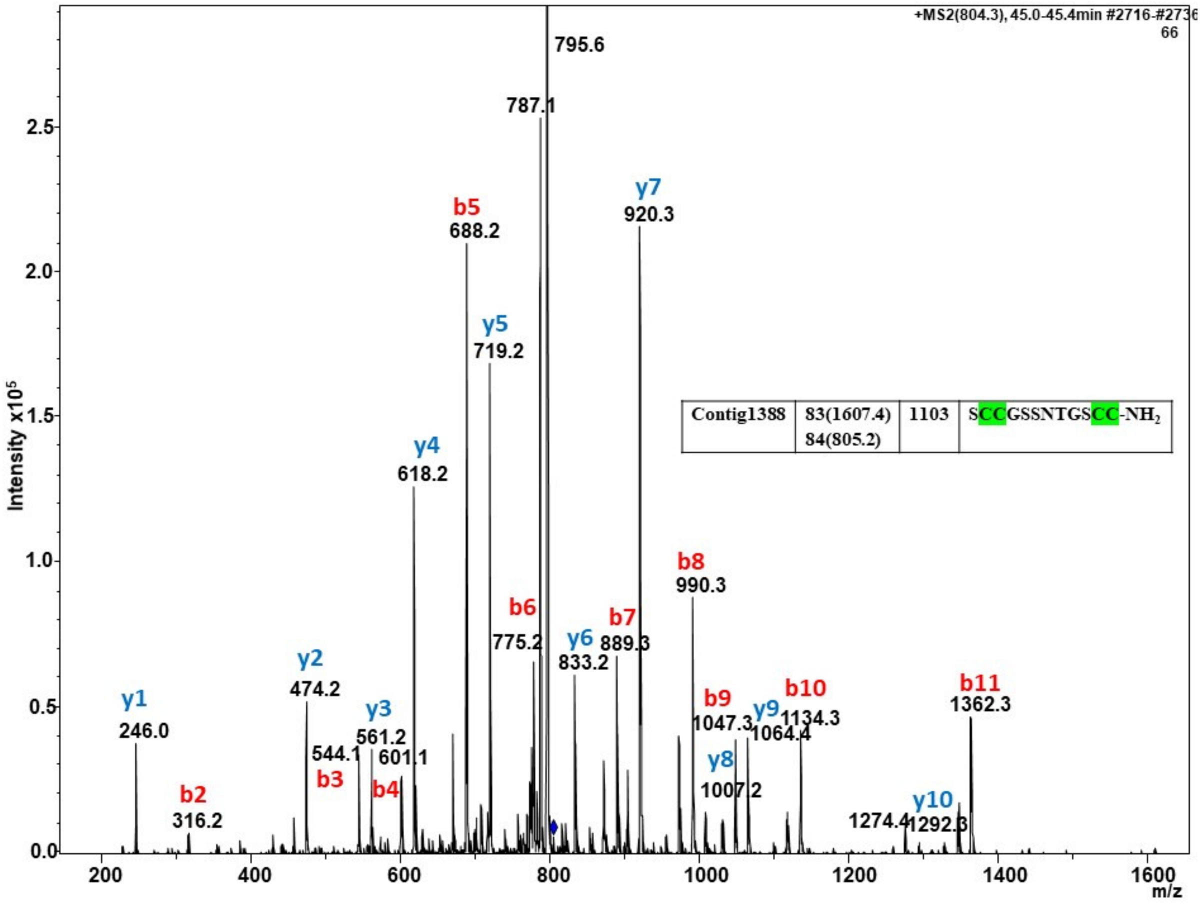

Figure S6. Native reduced alkylated spectrum of L superfamily conotoxin showing 504 Da increase in mass confirming four cysteine residues.

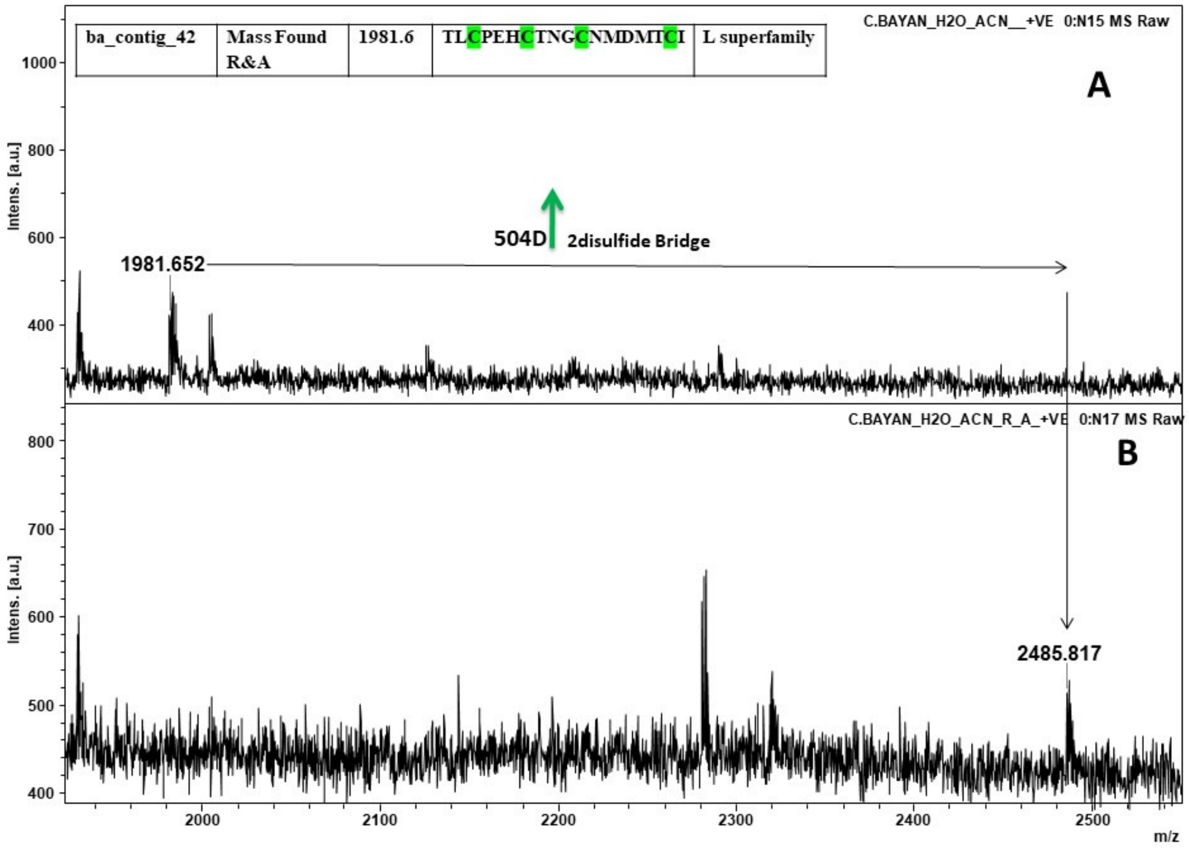

Figure S7: Collision-induced fragmentation of P- superfamily conotoxinba9a from *C. bayani* illustrating arrangements of 'y' and 'b' ions from the parent ion (reduced and alkylated) 1255.50 [M+3H]$^{+3}$

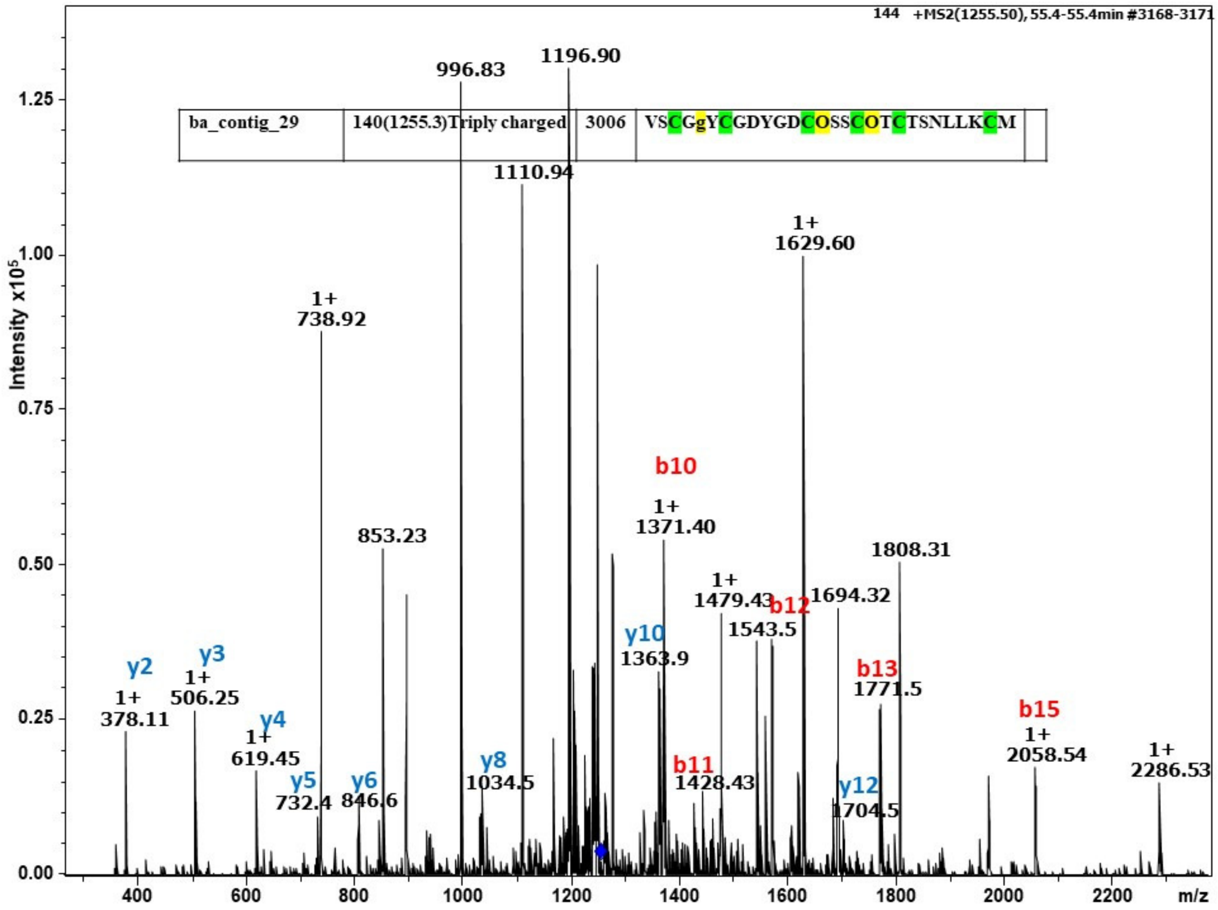

Figure S8: Collision-induced fragmentation of M- superfamily conotoxin ba3a from *C. bayani* illustrating arrangements of 'y' and 'b' ions from the parent ion (reduced and alkylated) 1141.61 $[M+2H]^{+2}$

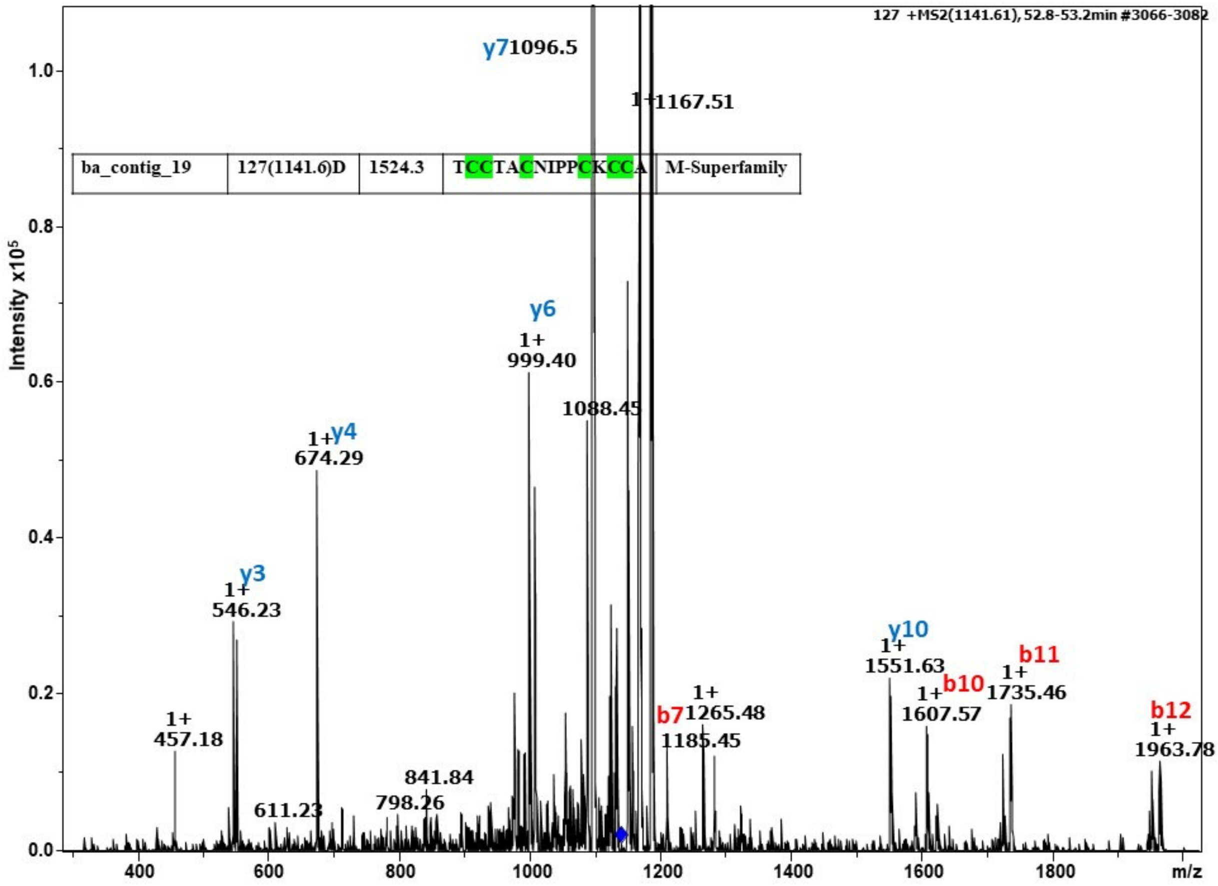

Figure S9: Collision-induced fragmentation of U- superfamily conotoxin ba2281 from *C. bayani* illustrating arrangements of 'y' and 'b' ions from the parent ion (reduced and alkylated) 1213.65 [M+3H]$^{+3}$

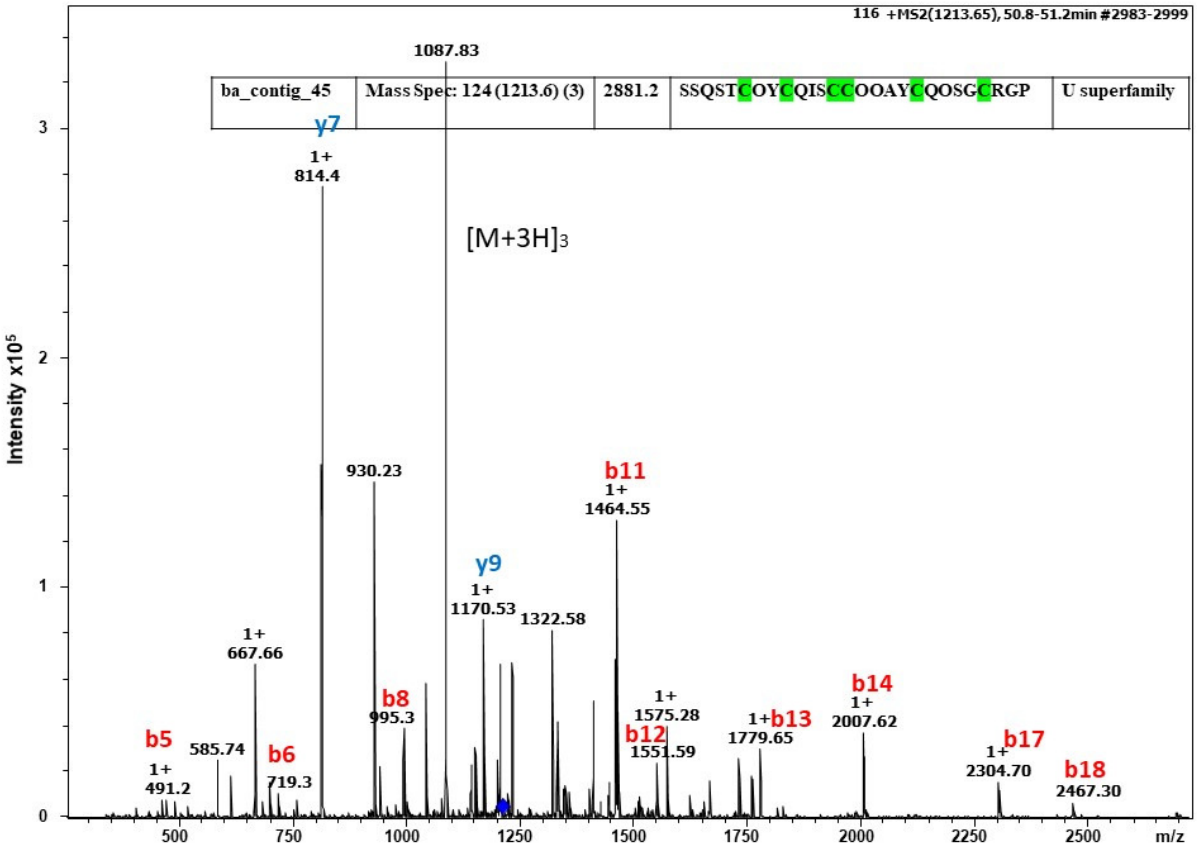

Figure S10: Collision-induced fragmentation of H- superfamily conotoxinba1560.9 from *C. bayani* illustrating arrangements of 'y' and 'b' ions from the parent ion 780.79 [M+2H]$^{+2}$

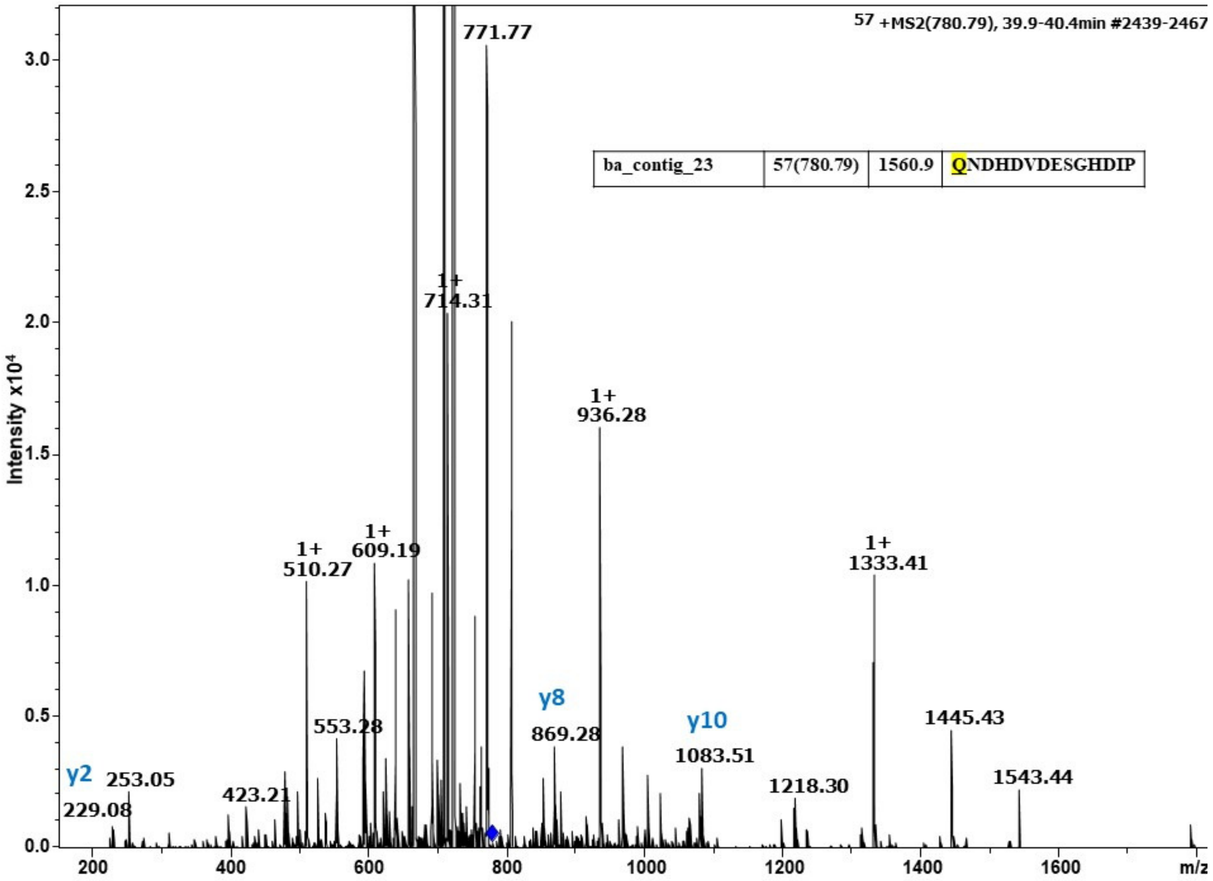

Figure S11. Collision-induced fragmentation of linearconotoxinba606.2 from *C. bayani* illustrating arrangements of 'y' and 'b' ions from the parent ion 606.19 [M+H]

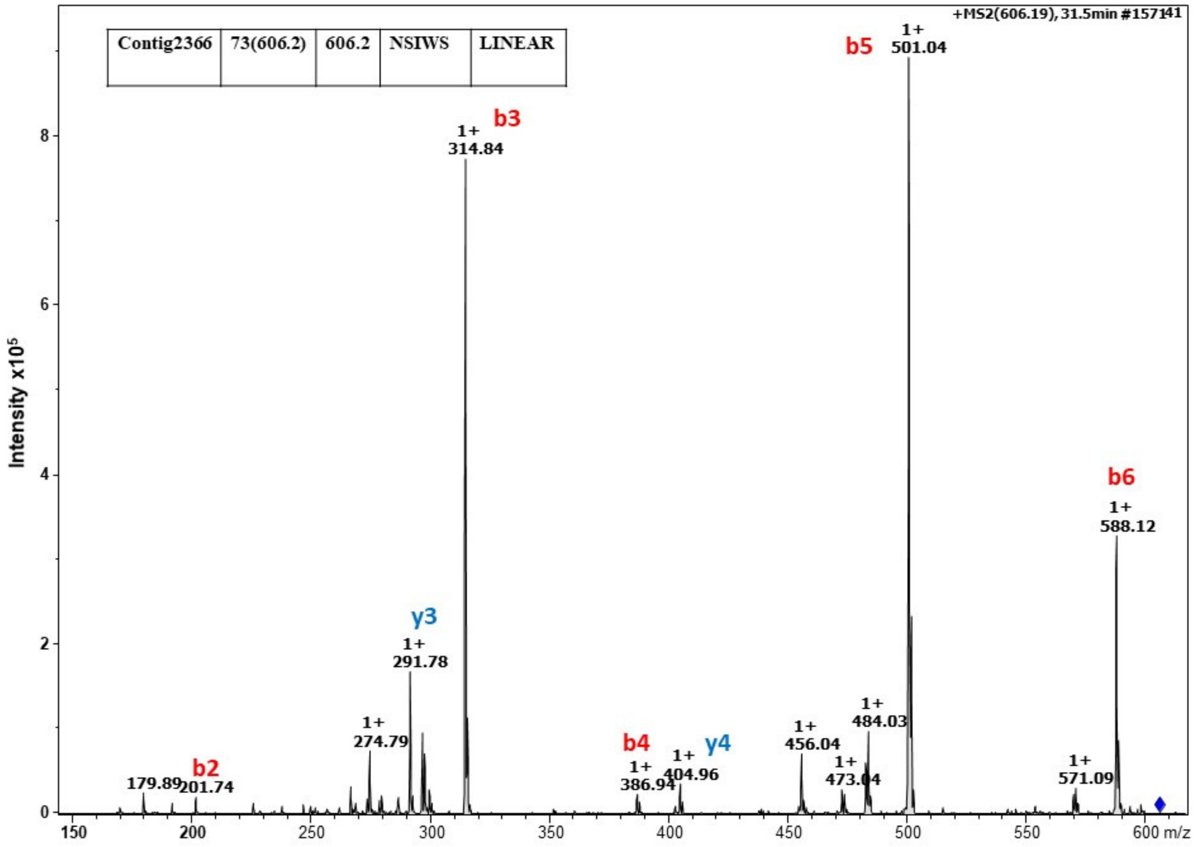

Figure S12 Collision-induced fragmentation of linearconotoxinba818.3 from *C. bayani* illustrating arrangements of 'y' and 'b' ions from the parent ion 818.27 [M+H]

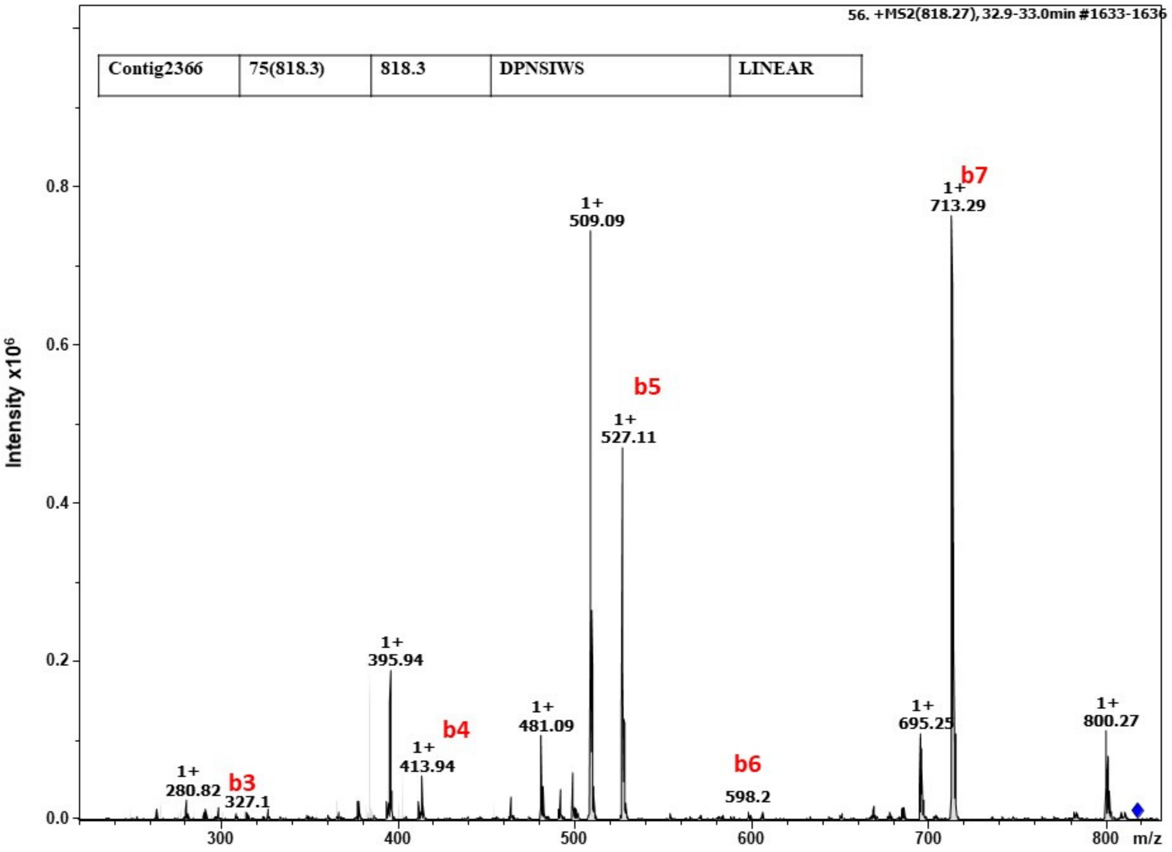

Figure S13 **Collision-induced fragmentation of linearconotoxinba731.3 from *C. bayani* illustrating arrangements of ‘y’ and ‘b’ ions from the parent ion 731.28 [M+H]**

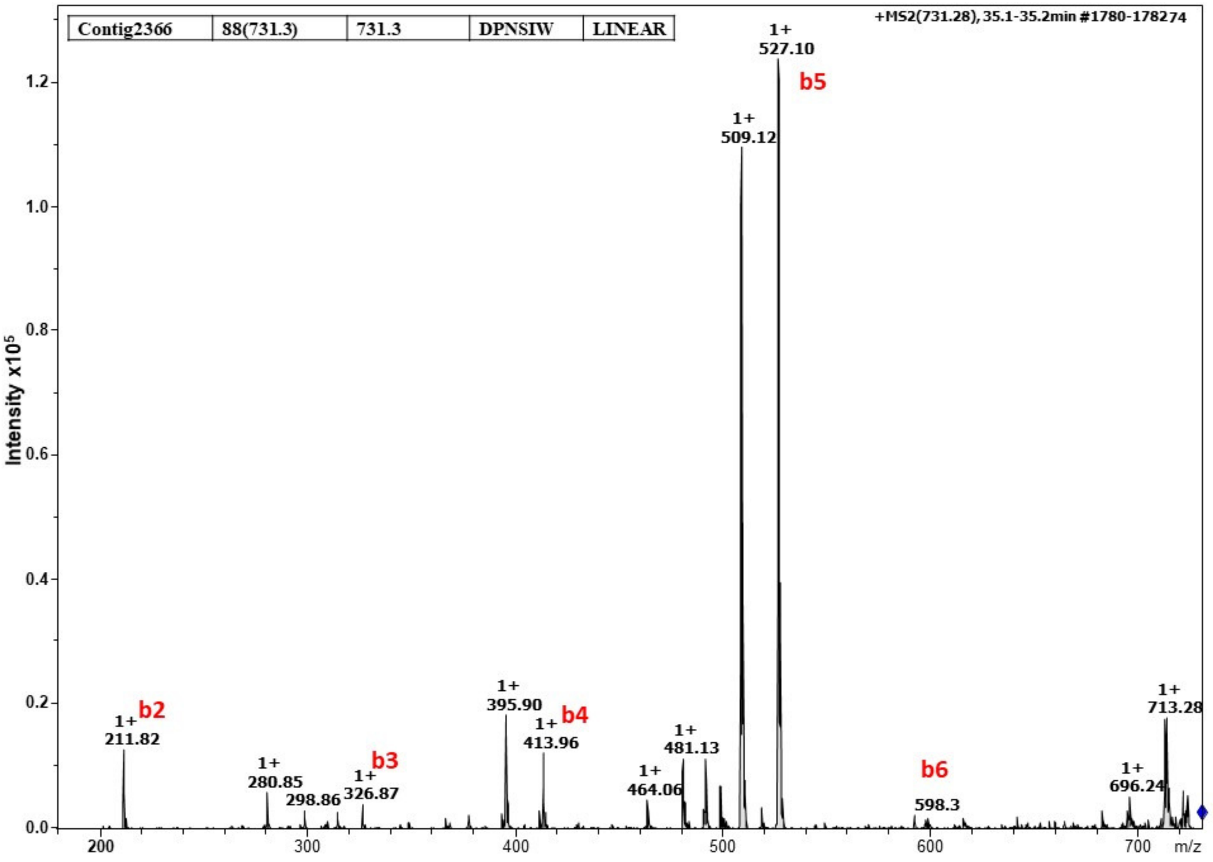

Figure S14 Collision-induced fragmentation of linearconotoxinba648.2 from *C. bayani* illustrating arrangements of 'y' and 'b' ions from the parent ion 648.29 [M+H]

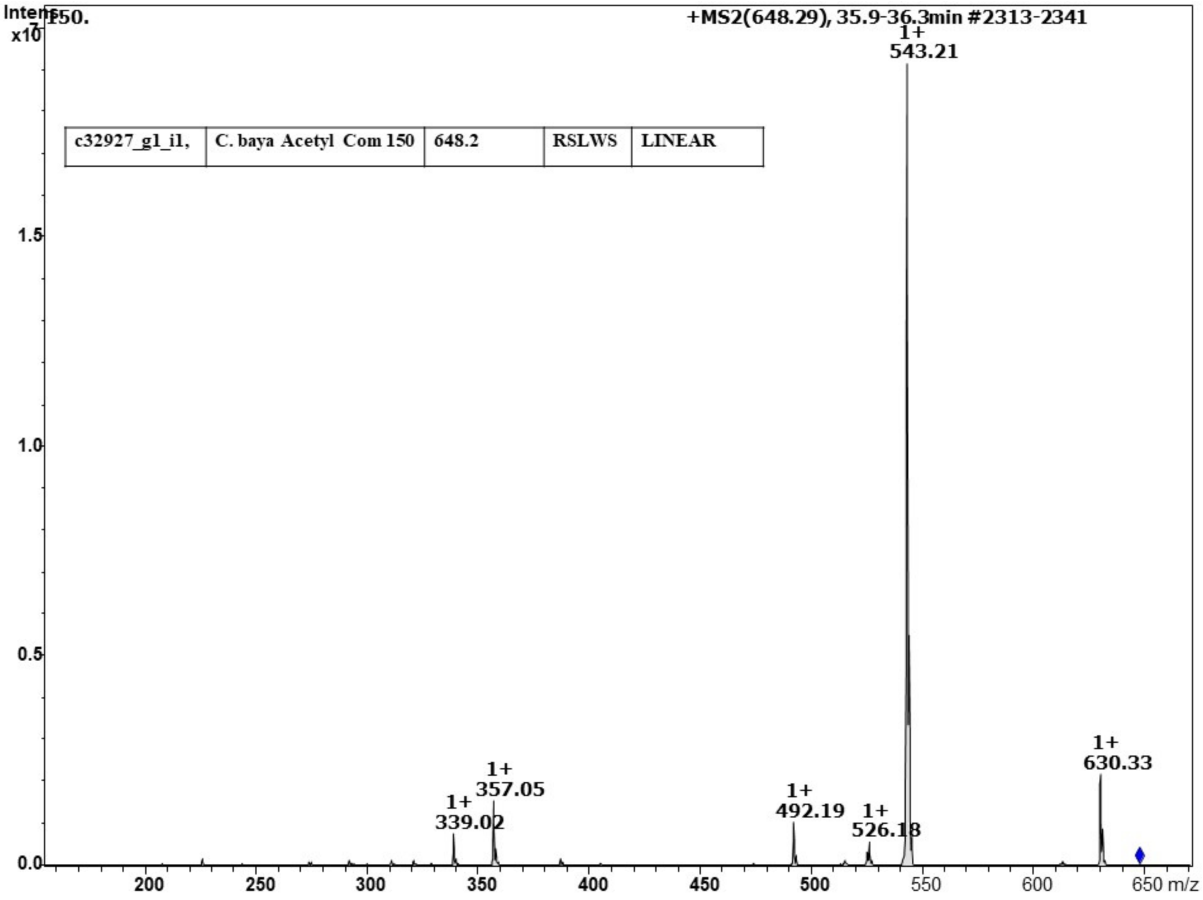

Figure S15 **Collision-induced fragmentation of linearconotoxinba745.2 from *C. bayani* illustrating arrangements of 'y' and 'b' ions from the parent ion 745.27 [M+H]**

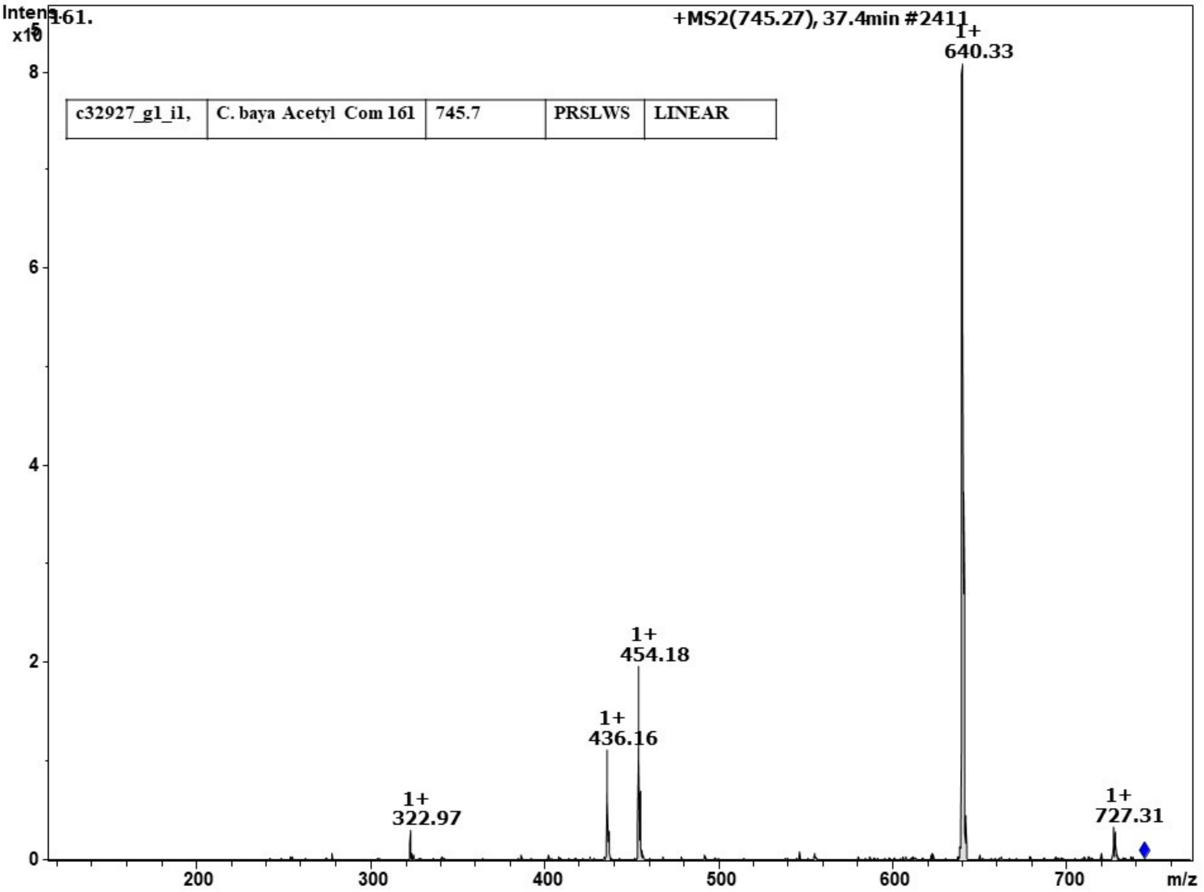

Figure S16 **Collision-induced fragmentation of linearconotoxinba561.1 from *C. bayani* illustrating arrangements of 'y' and 'b' ions from the parent ion 561.17 [M+H]**

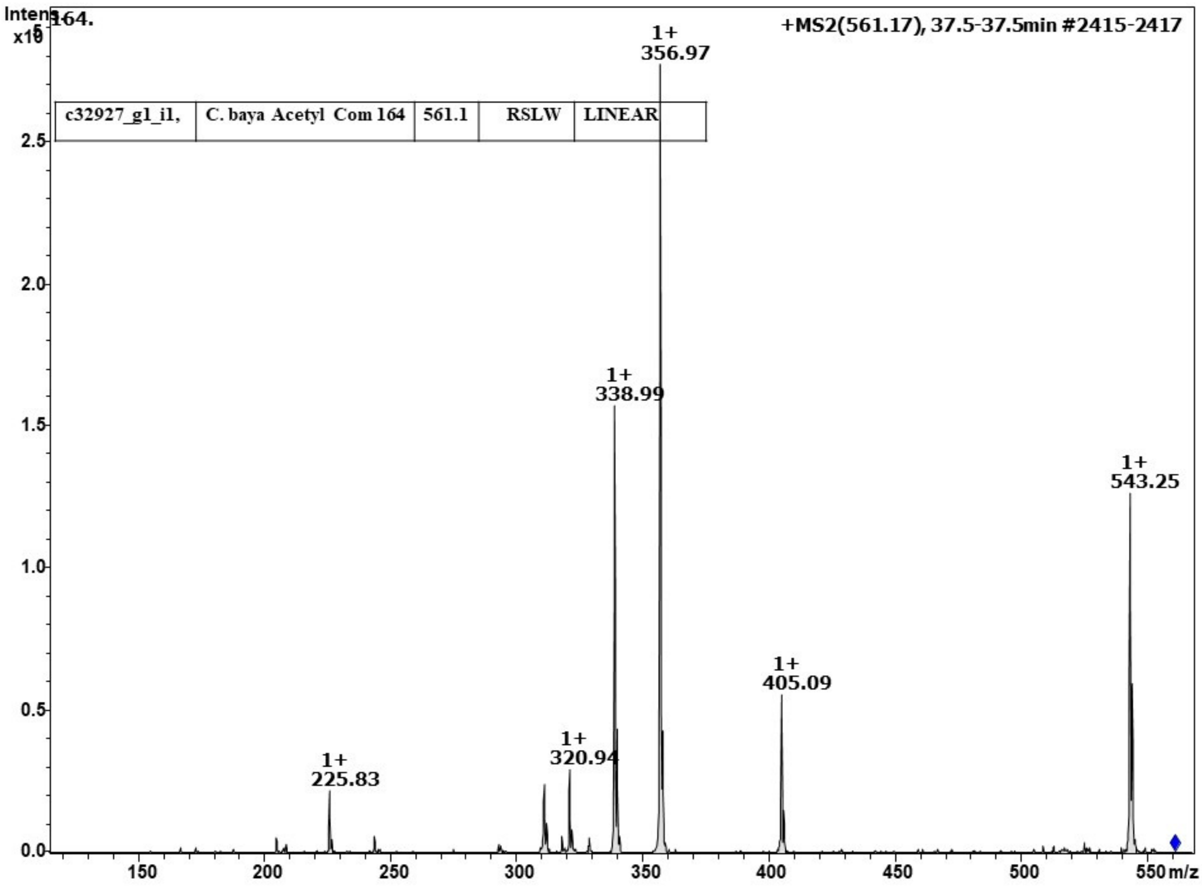

Figure S17 **Collision-induced fragmentation of linearconotoxinba416.9 from *C. bayani* illustrating arrangements of ‘y’ and ‘b’ ions from the parent ion 416.99 [M+H]**

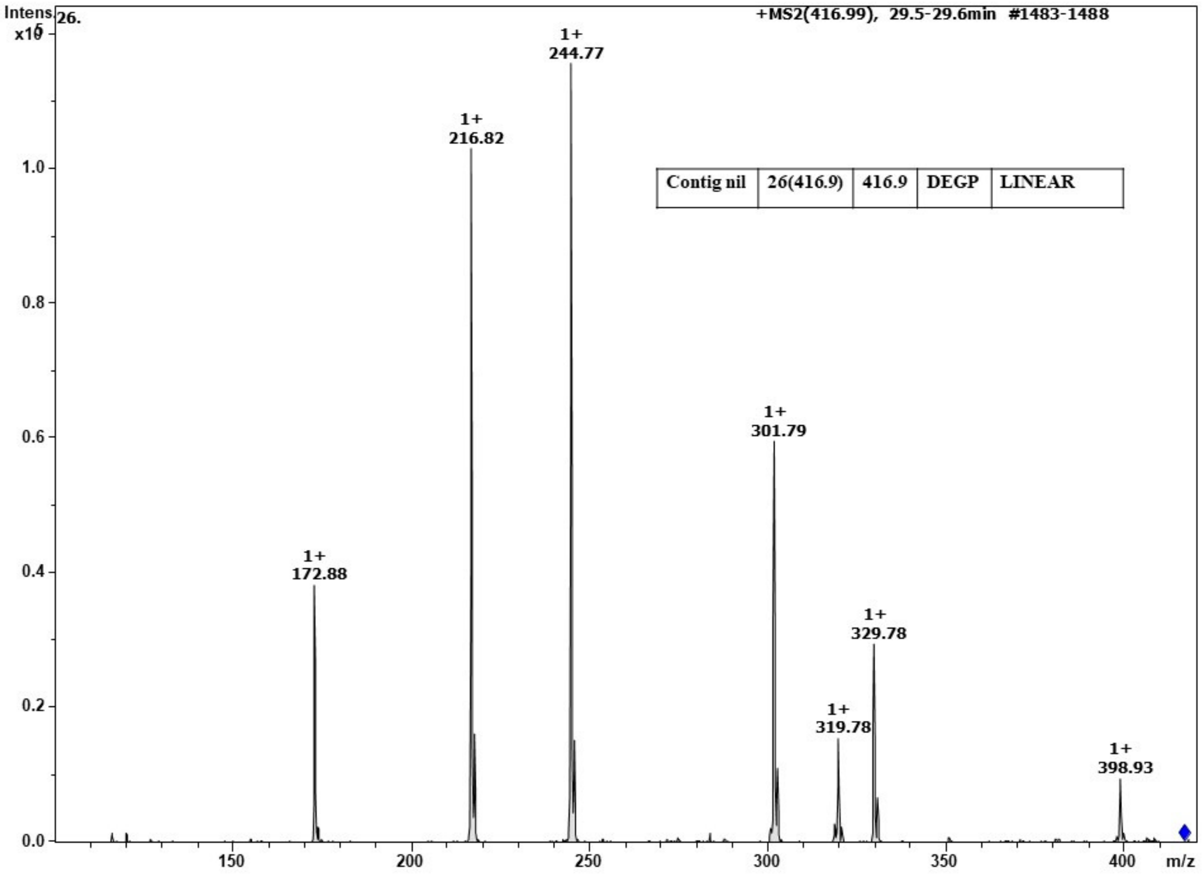

Figure S18 Collision-induced fragmentation of linearconotoxinba534.2 from *C. bayani* illustrating arrangements of 'y' and 'b' ions from the parent ion 534.26 [M+H]

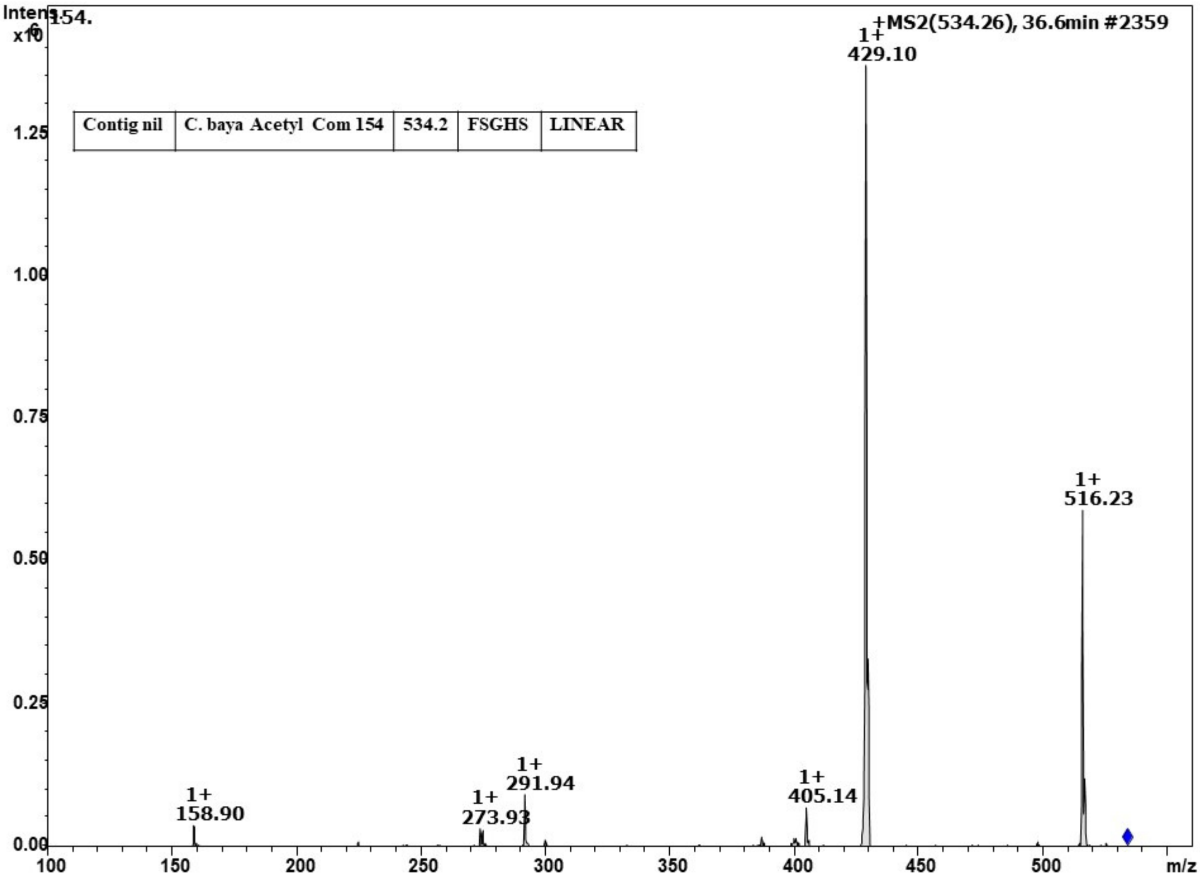

Figure S19 **Collision-induced fragmentation of acetylated linearconotoxinba774.7from *C. bayani* illustrating arrangements of ‘y’ and ‘b’ ions from the parent ion 859.24 [M+H]**

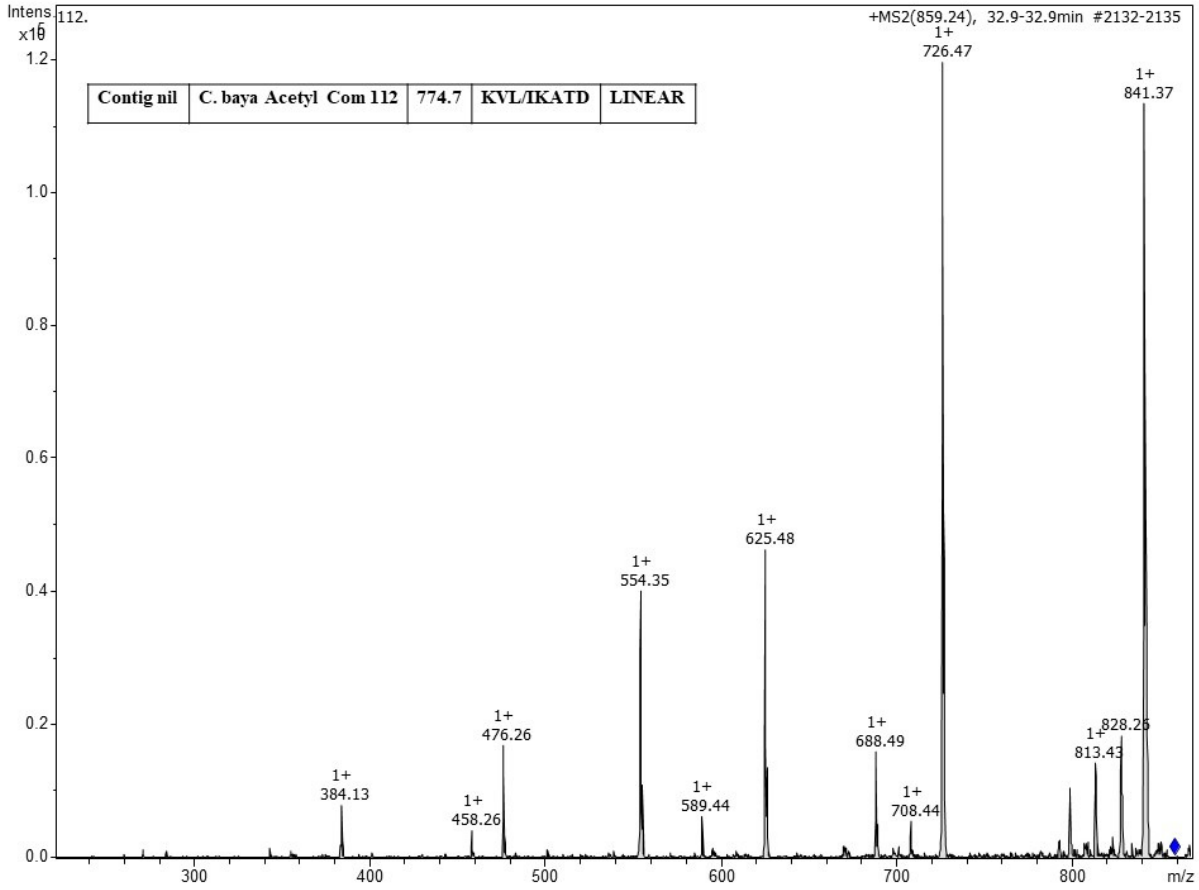

Figure S20 Collision-induced fragmentation of acetylated linearconotoxinba558.3 from *C. bayani* illustrating arrangements of ‘y’ and ‘b’ ions from the parent ion 643.37 [M+H]

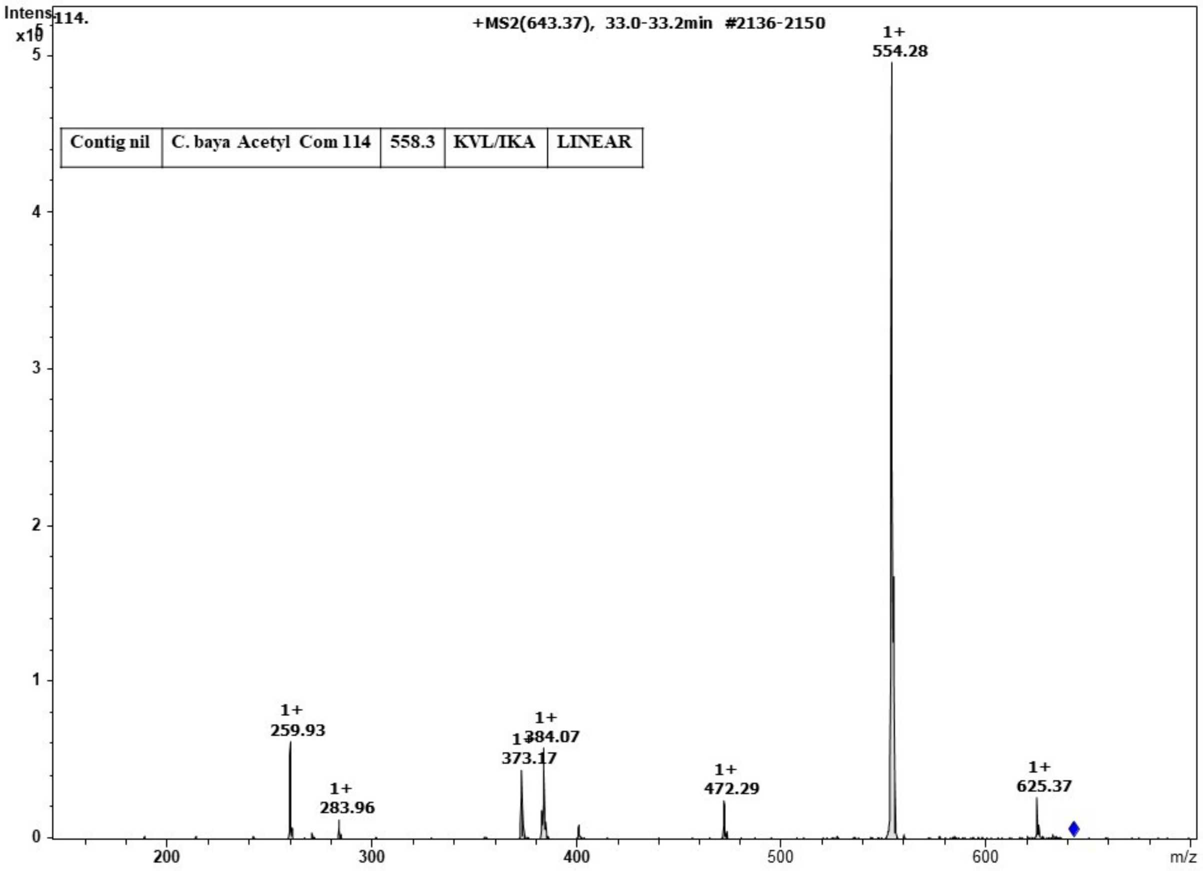

Contig nil	C. baya Acetyl Com 114	558.3	KVL/IKA	LINEAR

Figure S21 **Collision-induced fragmentation of linearconotoxinba998.2 from *C. bayani* illustrating arrangements of 'y' and 'b' ions from the parent ion 998.29 [M+H]**

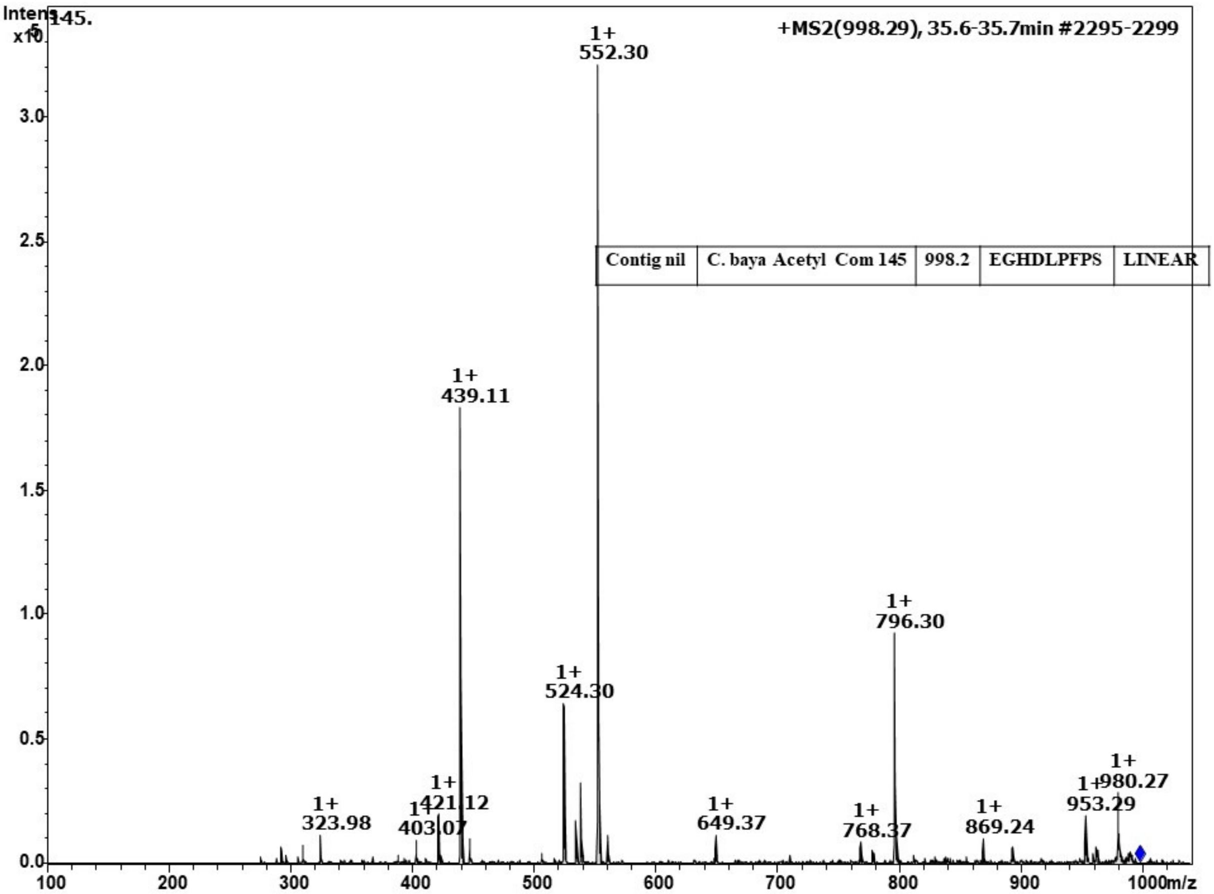

Diversity of conopeptides and conoenzymes from the venom duct of the marine cone snail *Conus bayani* as determined from transcriptomic and proteomic analyses

Rajesh Rajaian Pushpabai[1], Carlton Ranjith Wilson Alphonse [1], Rajasekar Mani [1], Deepak Arun Apte[2], Jayaseelan Benjamin Franklin [2]*

[1] Centre for Molecular and Nanomedical Sciences, Centre for Nanoscience and Nanotechnology, Sathyabama Institute of Science and Technology, Chennai, 600119, Tamil Nadu, India.
[2] Department of Marine Conservation, Bombay Natural History Society, Hornbill House, Dr. Sálim Ali Chowk, SBS Road, Mumbai 400 001, Maharashtra, India.
[2] Email: b.franklin@bnhs.org; benkutti@gmail.com
* Corresponding Author

Supplementary Table 1: Conotoxins sequenced from *C. bayani* venom duct transcriptome analysis with their possible cysteine frameworks

Contig	Sequence derived from NGS	Cysteine Framework/Note
**O1 Superfamily**		
ba_contig_1	MATHRYGRLRQKKREEHRRGMVRRQTSFRSRRCWCTIGYSRK	-
ba_contig_2	MKLTCALIVAVLFLTACQLIATDDSRGMQKHLAKRSRAKMLNYRLTRSCDPPGYECERLENNCCDACKIRENNPNVCSNE	VI/VII
ba_contig_3	MQNSKGAKSTRDCSPSGDGCHTRTCCPGLRCSGPQQAQVCH	VI/VII
ba_contig_4	MKLTCALIVAVLFLTACQLIATDDSRGMQKHLAKRSRAKRLNYRLTRSCDPPGQECERLENNCCHACKIREKNPNVCSNE	VI/VII
ba_contig_5	MKLSVVIIAALFLTACQLVTARNHARYLWESPRRKMQRTWEGKEVQPCKGPGSWCGGEEVPTECCEVCVFGWCT	VI/VII
ba_contig_6	MKLTCVVIVAVLLLTACQLITADDSRGTQKHRALRSTTNLSMSTRCKPPGSKCTPTVYNCCTSCNPYSHKCRTPWG	VI/VII
ba_contig_7	MYLLLGWVGGSGPYCSFMFMEHTFPAEWYQQGCRLYVGCVTVSCCLSYVSDCLRLLPLSLVLSLHCIGNW	VI/VII
**O2 Superfamily**		
ba_contig_8	MRGSRQCTPKGQPCEEDGECCSNLECKCFTRPDCTSGYKCKD	XV
ba_contig_9	MEKLTMLILVAAAVLSIQVTAGGDGEKPLMGRITRNAANGLSALMRGKRCKGYHAECERDSECCSGDCHCLNAADCAGFTHKCR	XV
ba_contig_10	MEKLTILLLVAALLMSTQALIQGGGERRKRVTNKCGPKPVAECWWDDECKGWSNYCGQSPEECCSADCAFYCKLW	VI/VII
ba_contig_11	MKKLIILLLVAAVLLSTQAWIQSDGEKRQKVKVKFLSKRKPSVMSWWGSKCNIFLMGCKVHADCCSNNCEGHCRLW	VI/VII
ba_contig_12	MEKLIILVLVAAVLLSTHVLVRGEGDEPADRNAVPRDNQDGMSGKFIRVLSGTCCMGYDCWCPRKPV	-
ba_contig_13	MSTQALIEGDGEKKGQKAKINFLTARNLLGNKKARGRCIGLSNYCGPWNNPPCCSRWICESRYCDFPNVLS	VI/VII
**M superfamily**		
ba_contig_14	MMSKLGVLLTICLLLSPLTALPLDGDQPAERLQDDNSAAQNHWFDHVKRCCPWPCNNGCEPCCG	III
ba_contig_15	MATFQLDAERPAERYLGNKQNLNRDERMRIISSTLKQRDCCKEEWCDGGCYCCV	III
ba_contig_16	MSNLGVVLFIFLVLFPMTTFQLDGDQSADRRADERGQGLTEQYRDLRGLKRSGGLPNSIWSKC	-

ba_contig_17	MEYADWLANTYQNVNAISLGGKRFVMLKMGVVLFIFLVLFPMATFQLDAERPAEHLGNKQNLNRDERMRIISFALKQWGCCDTGWCDAGCGCCV	III
ba_contig_18	MSKLGVVLFIVLVLFPMATLQQDGDQSADRRADERGQGLTEQYRSLRALRLSGADPNSIT	-
ba_contig_19	MSNTATWTFSIITSQTFPGLITPPARRQETVNATATPRADSHSYVKKGGKRFVMLKIGVMLSIILVLFPLATLQLVAERPAAERYAENKQDLNPDERRNYLVDLGVER TCCTACNIPPCKCCA	III
ba_contig_20	MSKLGVALFTFLLLFPLATLQPIGGQLADRNAEPRAGNPDGMYGFLMRIWNRDPRDDDDDDCPWCG	-
ba_contig_21	MSNIGIVVLIFLVLFPLATAQLDADQPADLEGEKRGGWLPKMYQQMKEVLNRGTRCGGYCTDDVCCKRSFLKGMALIAAEDKRRRTAMGQ	-
ba_contig_22	MMWKLGVVLLIFLVLLPLTAPRQDGDGMAYTGRHVLHRMKNALKITKRDCGERDEPCCVNSSGVKYCESPWSCMHTTLLCEQN	XXXII
**H Superfamily**		
ba_contig_23	MKTSGRLLFLCLAVGLLLESQAHPIADAEDATRNVGSDGTSVELSEILERGQDSSAEKGQRQNDHDVDESGHDIPFPS	Linear
ba_contig_24	MKTSGRLLFLCLAVGLLLESQAHPIADADDATRNVGSVETSVELPEVLERGQDSSAEKGQRKATPYDHRYGVPWPTSADIPDYRSLSGGR	Linear
ba_contig_25	MNPAGRLLLLGLALGLLFESLGKPMADDVHAERDTDPGDKAPRAISAERADVPCGDGTCTFGCCENGICKELNCLDVSNTESSWKRWSLSGSR	VI/VII
ba_contig_26	MSASGRLLFVCLTLGLVFALLGNPIPDVGDAARDAGPDGGSLERSETIEGRQATLSERNTRNRDVPACPEPPAPNPPENTNCENPVC	Odd cysteine
**G Superfamily**		
ba_contig_27	MNCLQLLLVLLLISTITALYPDGWATLRRGKTIRRMSNLLNIQKRKCPDNCPSTCPERDECCDGDSCLYNSYMRKYYCYDCGSGGPN	XIII
ba_contig_28	MKCLQLLLVLLLISTIAALYQDGRATQRRDGNIRTMSDLLNIQKRECSSDCVAECPNGNECCDGDLCVYSSVLETYYCIGCGSGGGE	XIII
**P Superfamily**		
ba_contig_29	MHLSLARSAGLMWLLLFAVGNFVGVQPGQITRDVDNGQLADNRRNLQSLRKPMTLFKSLNKRVSCGEYCGDYGDCPSSCPTCTSNLLKCM	IX
**I-1 Superfamily**		
ba_contig_30	MMLSVTFLLILMILPSVTGEKSSEHTLRRLKLARIFRGGCSEIGEGCGHHFDCCGDMCCFHGTCAVSATGLGCDHF	XI
ba_contig_31	MSRSGMALLVFLLLLSLVTNLQGKGEGQTMHQNKHRQTVRKLMTLRRTQKRNACELDSSTGDDCTGTQICCNEPGSMSGECKETDECPDRRR	VI/VII
ba_contig_32	MKLFMTFLLLLMILPLCQSSGLRQLLATNRFGSKDKPRSAVSKRCSGNPCSTERKCCKGYFCGEGKCLSRQRGTFRNGK	VI/VII
ba_contig_33	MKTVAVFLVVALAVAYGQFFCPSSKDEPLNCIETMASTPTCMKSTADESLSYACGYCGKKKETCSGDKVPVSNYNCQIRKIPNPCGGPAL	XXII
**I2 Superfamily**		
ba_contig_34	MMCRLTSLCCLLVIVLLNSAVDGIPCNEGGGWCSTHMWCCDLFHVCCDSPGQAVCKTDSECSWPHIPQNRGALYTRFFRR	XI
ba_contig_35	MVRRTSVSCCVLLVIVLLNLGSAIMVQKKQKIVCDQEEMFCTIDGECCLHECCLGKCSSPCIPGKRALRDDLLSFIRQR	XI
**I3 Superfamily**		
ba_contig_36	MKLFLATVFILMLLSLNTGAETSDNRATRSATALRDRLLRPKRCQAQYENCWKNSQCCEEYCCTGASYCDHSIGRCDMGK	XI
**F superfamily**		
ba_contig_37	MQRGAVLLGVVALLALWSLAAADLYDWNDQDVRYMALYTQALMTVCARANKYIDNPWSMLSLEAFKEKSRYHAMVNEMVVCLNHYLQKRHEIP	-
ba_contig_38	MMQRGAVLLGVVALLALWSLAAADLYDWNDQDVRYMALYTQALMTVCARANKYIDNPWSMLSLGAFQEKRLYHGMVNEMVVCLMNYLERRHEIP	-
**B1 Superfamily**		
ba_contig_39	MQLYTYLYLLVPLVALHLILGTGTLAHGGALTEDRSADAIAQKPEPALLQRSAARSTDDNGKDRSTERKRTTNKRRNAARRSQAEEMIIHK	Linear
**B2 Superfamily**		
ba_contig_40	MLRLIIAAVLATACLAFPQRRDGLPGEAANLKAFGQDMQGMQAMPGVMSAPLANMQPMQAMPGQFLPNFGMGFKRAADENLEKRKHHSKFQNE NKSPFDSSADSLLGNFDLGKFLQENPDNIPFANTENANPADLGNFEPNAEGSKEGHFRFFDHQQ	Linear

**Y Superfamily**		
ba_contig_41	MLKMPVLLLAILLLLPLATAQDDKRSQAHATQRRDAPPCAGSQSPCDEPAGQSCCGTLKCVSNRCCPTTDGC	XVII
**L superfamily**		
ba_contig_42	MKLSVMFIVALVLSLSMTDGLPRRAENGGRIFRQHSPDSMDPQTRQIKTRTLCPEHCTNGCNMDMTCI	XIV
**A Superfamily**		
ba_contig_43	MRCLAFLVVTLLLVTAMTTAARLGPAYDGWDAAADDEASDPIVLAVRDGCCSTPPCIANHPELCG	I
**T Superfamily**		
ba_contig_44	MLCLPVFITLLLLVSPSAALPVESELQRDLTQDSPKDFRIREPLLLSKMFDRSCCGSSNTGSCCGRYQRGS	V
**U-Superfamily**		
ba_contig_45	MNRMGFFLMLTAAVLLTSLVCTEATPADESKVKRARWSRIEGSRLFRHRLPKSSQSTCPYCQISCCPPAYCQPSGCRGP	VI/VII
ba_contig_46	MNRMGFFLMLTAAVLLTSLVCTEAAPADEAKMERAQQSNRDRSRNPEKRCVDCRPGYKCCGVCTMNQCTGREIPKE	VI/VII
**New S Superfamily**		
ba_contig_47	MSLLILVLLAISALTLHTDSTQGHDGGTDKSSRPMARAARDHVSPALFRKFRARANVRTSRIKRVQEDFPGGEEEEENDEWSDGRLQALLNKKIQNKFFKFQNLHKLIH	Linear
**Contulakin C-Superfamily**		
ba_contig_48	MQATFLMMAMMMVWTAGPLSEGGKLNDVIRGLVPDNLTPRHVLHLTSIHHHDSVPGQICIWKVCPPSP	-
**Conkunitzin**		
ba_contig_49	MEGHRFAAVLILTICMLALGAGAFRLHGSRAEECSRAEALAKCYLPKDPGPCNARKPRYYFNRYLNTCQEFIYGGCHGNANRFYTMEDCLGCCLLSVCRQPAE PGLCNAYMERYYFDLDSYDCKPFIYGGCNGNDNKFHTYNECYGRCGLE	-
**Conodipine**		
ba_contig_50	MKMLESALWILAVLALPRIAAQDSRTTELCKINSNGCSVQFDWVPCQEHFLPACDIHDNCYFCGAHFSLSRLNCDDAFLSDMIALCADGTDEESDCPAKRKRRE ASSMSTTPVRQLQLLEKLMGRNSLSDHDPRRPLPRSSTCTGWAQTYYNFVRWFGASNYNETPDATYCSDYEECMPEV	-
ba_contig_51	MKMLASVLWTMAALGVTWLLAEDSTSEQSCKRFSNGCSTPLPLPCQEYFRPACDRHDSCYQCGAHFGINRKQCDDAFSDHMHALCDELGLLGMCPARRK RQVASGRATPIARSTLLKRALPQKSSLNREARVFFAPTFCHEWATTYYSVVRMAGAGLFFETIFDPADCQGLEACMPDH	-
ba_contig_52	AVMMMMAMPVKADQCDDHPTVNGCTTPSFLDLQHEKTFTPACNRHDVCYGCGVKYGVTRTQCDEAFLRDMKEACRLERRRRKLTVNVNMDCPDMAN AFHTAVWAFGHSHYVDAGTPNSDCLEELDKSCLP	-
**Conopressin/ Conophysin**		
ba_contig_53	MGRLTMALCWLLLLLLTTQACYITNCPRGGKRDVDDGLGVRPCMFCSFGQCVGPHICCGAGGCEIGTLEASTCHEENENPIPCHVFGDRCLLKHPGNVHGN CVSPGVCCTDDTCSMHVGCL	Odd cysteine
ba_contig_54	MTRSALQMGRLTLVLCLLLQLVLVTQACFLGNCLNDGERDVDGREAMRPCKYCSFGQCVGPQICCGDRGCEMGSEEANKCREEDEDSTPCQVFGWPCTLN NPGNTNGKCVANCIGICCVTDTCVVSSECQKESKSGIRVGCQRS	Odd cysteine
ba_contig_55	MKCSVLQMSRLSWAMCLMLLMLLLLGTAQGCFIRNCPRGGKRAVDAVQPTRQCMSCGPGGVGQCVGPSVCCGLGLGCLMGTPETEVCQKENESSVPCAIS GRRCGMDNTGNCVADGICCVEDACSFNSLCRVDTDQEDSVSARQELLTLIRRLLVNRQYD	-
**Con-ikot-ikot**		
ba_contig_56	MTPIVFVTVVMAATVIGSTPLQEQELNRNDRDIQECCANKANECLRNDGCYQSQEESCAKRCYYTDTSSCGDQADVVCCFDYQYCMTECLFPHQDLYPEMLG DCYDYCKVYEQC	-
ba_contig_57	MAMNMWMTTSVFLVAVTATTVIGSTPSQEQERERRTDVDLCCATRIYYCLKDNGCLPQRECTVACDVPDDCANCCQAYLDCAMSCIWAYELPTGTEEEDPLR DCHNQCKDGC	-
ba_contig_58	MNMRTTISVLAVAVMATTVTASPLLQDQERDTDDRKCCAIALYQCLRDEGCLESGSSCQITCTFPHDCFEICCHDYMLCVYNCLHGREEGEDIMRVCHTACTDTVCSE	-
ba_contig_59	MWMTISMFVVVVTAATVVGSTPLEERQPGDCCHLVFYECAAEACTFTDDPGCTEMCWDVATDVCGGDPDSGFCSSFFGCFNDCVFTESGPHFLCYELCKMV PCWLVK	Odd cysteine

ba_contig_60	MTMPVTLSLLVTIAMAATVLDSTLLRGNDPSCEDQVYHECCRHEMHSCAEECLSASDMDFCWEPCTQSAAVQCGRQTSSACCRPFLECFSACLNDGDVLYDC WIRCRHVPC	-
ba_contig_61	MTMNMSLTLSLFVMVVIAATVVGSTPLEGKDVNCEGLAYHECCRNEMRICILECSSVWDADSCWDYCYSSAATNCECHPRDDCCPGFLESYSSCLFHDGLEE YECWEQARYVPCW	-
**Conoporin**		
ba_contig_62	MGVPFPALKTMVTVFLLLMGNTSPVHSAGLGSTISVAVASIASSVVSAGTTLAGTTLSGLADPNFSVSCAIQVENWTRYALMYPTVRTRGNPAVTTVPTAIL PTKKEAFALRKPRHTATGVAGTVSWELQGAKLRCVLMWSAPYNFNHYSNWMGVGMTDEGLVNVASGNTWWDQMYYGSNGGNLTFQRGEFYYDLKP VIYRNDMFEISGTMTNIHKAEIKVVIRPTTDNWKDLAVPIQALLVMEDEEVCSMTTEGK	-
**Insulin**		
ba_contig_63	MARRLGILVALGLLLLLSHASDDHYCDPNAPPEYPQGICGPDLPEMVSLACSLAGGKRHSERETVGQLKKRGSIASLLKTRAKRVIEAQGIVCECCIHQCIFEEFSEYCWVW	-
ba_contig_64	MSTSPCFLLVALGLVLYAWQACLGDEHTCDSSSTPHPQGKCGSELSEHREELCEIEESLHGGIDDARKKRGRALPLKKRRRFLLKARAKRNEALPLDRARRGIVC ECCKNHCNYEEFTEYCPPVTEGSS	-
ba_contig_65	MATGLLSPLLVTMLGVLLHVHVARAGLEHTCTLETRLQGAHPRGICGSKLPNIIHTVCQVMGRGYAGGQRQLRKRTSMINSDDMEADEGSVGGFLMSKRRA LSYLQKETNPLVMAGYERRGLQKRHGGQGITCECCYNFCSFRELVQYCN	-
**DGF**		
ba_contig_66	MKSTLFLTLLLAVVFLTFFTETDTTSIFKARAKRADSEEFPCAGTFADCRDQANGTTCCGDGACYGEVCYY	VI/VII
ba_contig_67	MKVVVVLLAVLVAASAAPQKRFFMQDIKNWIHQLQAAFNKAKDKFNELTSGLGVHFDRIVDLLIDQIDSGMTEAACIKVCETSSNKILGNASSMAGVVCA PVCTAALAKLEEVAG	XIV
ba_contig_68	MQKRWRWSVRETGHTPVLSCTVQWPFCCVFVNTSSIIAYTPRAVASVTACCVIRLLPAVYTRVLFCKPYCTRVLIVQSFFLLSLWL	Odd cysteine
ba_contig_69	MKFPTFVMVLMAAVLLANILKTDAMRSFRARVRAKRTLEEMFEACSGTFTDCRGQPDGTLCCTDGYCEGDVCYY	VI/VII
ba_contig_70	MSKLAIVLLIFLLLQLATNQHHPDERAVRLAKKNLKKFRSLAMGRRKDACNGTEDCEEDDDCDGCECVEVDAQGKCMEVTPPGR	IX
ba_contig_71	MQRSYVATSRMDFRRLVTVALLLTLVMSTDSAPADQTETGRVSLREGLENQFPCSTGRCACLPKDGSSSRYQCQSVGSSTAGCLDGKCVTEDQW	IX
**UGF**		
ba_contig_72	MGILTVFLLLVAVLVLTQVMVQSDQDKPLNRKRNLREAQFLRRVRGDKKQGEACEERSECAFGLICKNGACEHVSPGPGRALPDRAVPIRQRKPEIG	XIV
ba_contig_73	MPSFFYNTGTGQCERFVYGGCGGNANRFETKQECERKCQRIIYISNAVPSQNPACLQPRVVGPCRARMPRYFFHQSTQTCELFYYGGCGGNSNNFRTL EGCEGACVSGEAVQDVCALPKVAGPCFAAFPRFYFDKTAGRCKTFTYGGCHGNQNNFRSLRACRNTCPGN	-
ba_contig_74	MCAMKIGLIIYLLLIAFMNGDGSPGNTLYSRKGAGIASGMKRFQKIFLRAECGDCPEQPCCDGDKCMADPGYEPFCE	VI/VII
ba_contig_75	MMKTLCGNAVWGLLTVLLISMGKANGQNCGWFRKFDVQGCFNNFVVDFNQIFEPSYTEQLCSQVQPLEGCVGAVRSQCPHLAIFQGDKGLAIGTVVT SLSQLCVGNGGNVSTPASGGRCPGQKATFFRRLDRCHEPMQWNLQESQMCQPTSEIIACMETRLQEFRPCSWMTEFIADRAQFWIDFERALWFVKYECAGR	-
ba_contig_76	MGATLVTKLLLVAALLLGLCHEMAANPEAWDECYERMLMHMIAPTWSSMFKCHKILEYRKRAALVMKLKQLGVIHLDHGGWVVQQVKLLKYLGVDN	-
ba_contig_77	MEALTIFRLCLLVALTTSVVESGPLNDKVSDQEGECPVGGGRNPFVLCMRACLTTSTPYLCEHEYCKHCRGRYARVGHS	Odd cysteine
ba_contig_78	MSKRSADSAELSDEKQSSKRIQQKFRKTYSEKYPCIRGSIKGVHFARCTICFTDFTISHGGIGDVKRHVDSKKHLDASNSKECSSMVMTFFKPKEQDKKDG NKVIRAETFFAGFLVEHNIALSAADHAGHLFKQMFPESLMILKCTGVPCHKASYSDSLLEKCKAATAMSLAHK	-
ba_contig_79	MLSVFAWTLMTATVVVIAERQYCPVARETCYYDNDVCGKQEQSGSCSPRCNCKSGQLCSRDSDHTIAVVPGFINDRPIVRR	-
ba_contig_80	MSDCQSNARAMSGTSESTYKILCKCDATYEPSAPTNWKFICG	-
ba_contig_81	MLFVFTVVWVLTTVMMMTDVTFQSTCNTDNKPSCSEDTRLCGKKNSWGNCVALCKCPNQQACTTDTDHKVQVKRGPFQSTETYYTCKDVSTMSDCQ SNARAMSGTSESTYKILCKCDATYEPSAPTNWKFICG	-
ba_contig_82	MPMSMWMTISVFVVAVMATIVIGSTPSHVQERGRRSEVNKCCAMRVYTCLKDNDCIEAQAQCDGPCDVPGTDWREMPCLQRSKQLREYTPRLRPV RSFTSHHGHPSEIEPRHHISTALYLSPWTPLRNRTTSSHLYSTLPLTMDTPQK	Odd cysteine
